# Whole-Genome Quantitative Trait Locus Mapping Reveals Major Role of Epistasis on Yield of Rice

**DOI:** 10.1371/journal.pone.0087330

**Published:** 2014-01-29

**Authors:** Anhui Huang, Shizhong Xu, Xiaodong Cai

**Affiliations:** 1 Department of Electrical and Computer Engineering, University of Miami, Coral Gables, Florida, United State of America; 2 Department of Botany and Plant Sciences, University of California Riverside, Riverside, California, United State of America; Nanjing Forestry University, China

## Abstract

Although rice yield has been doubled in most parts of the world since 1960s, thanks to the advancements in breeding technologies, the biological mechanisms controlling yield are largely unknown. To understand the genetic basis of rice yield, a number of quantitative trait locus (QTL) mapping studies have been carried out, but whole-genome QTL mapping incorporating all interaction effects is still lacking. In this paper, we exploited whole-genome markers of an immortalized F_2_ population derived from an elite rice hybrid to perform QTL mapping for rice yield characterized by yield per plant and three yield component traits. Our QTL model includes additive and dominance main effects of 1,619 markers and all pair-wise interactions, with a total of more than 5 million possible effects. The QTL mapping identified 54, 5, 28 and 4 significant effects involving 103, 9, 52 and 7 QTLs for the four traits, namely the number of panicles per plant, the number of grains per panicle, grain weight, and yield per plant. Most identified QTLs are involved in digenic interactions. An extensive literature survey of experimentally characterized genes related to crop yield shows that 19 of 54 effects, 4 of 5 effects, 12 of 28 effects and 2 of 4 effects for the four traits, respectively, involve at least one QTL that locates within 2 cM distance to at least one yield-related gene. This study not only reveals the major role of epistasis influencing rice yield, but also provides a set of candidate genetic loci for further experimental investigation.

## Introduction

Given the paramount importance in sustaining food demanding, great efforts have been made in large scale genetic research and extensive breeding programs in almost all rice (*Oryza sativa* L.) producing countries [1], [2]. Gains in rice yield in recent decades are mainly owed to advancements in breeding technologies including selection of cultivars with higher productivity and significant increase of agricultural inputs such as fertilizers and insecticides [3]. While global environmental degradation has limited further yield increase through more agricultural inputs, studying the underlying biological processes of rice yield, and transferring the knowledge gains into improvement in breeding and agronomic productivity have become the key for further increase of food production [4].

Rice yield is determined by several factors including the number of panicles per plant, the number of grains per panicle and grain weight. These component traits and the overall yield per plant exhibit continuous variation since they are influenced by multiple genetic factors named quantitative trait loci (QTLs) and other environmental factors. Genetic markers such as restriction fragment length polymorphisms (RFLPs) [5] and simple sequence repeats (SSRs) [6] have been utilized to identify QTLs for understanding genetic basis controlling rice yield [7]–[11]. A recent study on QTL mapping for rice yield derived a high density single nucleotide polymorphism (SNP) bin map from genomic sequences obtained using deep sequencing technology, and demonstrated that such high density SNP bin map enabled to identify more QTLs with higher location precision than the traditional approach based on RFLP and SSR markers [12]. However, these studies attempted to identify QTLs individually via single interval mapping [5] or composite interval mapping with a small scan window [13], which had limited power of detection, given that many agronomic traits are controlled simultaneously by multiple QTLs and influenced by environmental factors [14], [15].

Whole-genome marker QTL mapping employs a multiple QTL model that includes all available markers and evaluates effects of these markers simultaneously [16]–[18]. Such approach overcomes the limitations of the traditional single marker-based QTL mapping methods [16]. However, when genetic interactions are considered, a multiple QTL model can have a huge number of variables, which makes model inference very challenging. Early methods for multiple QTL mapping usually rely on Markov chain Monte Carlo (MCMC) simulation to fit a Bayesian model [16]–[20], which is computationally intensive and unpractical when a large number of markers are considered. Recently, more efficient and accurate methods have been developed [21], [22], which make whole-genome marker QTL mapping feasible. With whole-genome marker QTL mapping considering main effects and interactions of all additive and dominance effects simultaneously, contributions of numerous genetic effects to rice yield can be assessed.

In this study, we applied our empirical Bayesian least absolute shrinkage and selection operator (EBlasso) method [21], [22] to whole-genome QTL mapping for an elite *indica* rice hybrid, Shanyou 63 [7], [23]. Our EBlasso model includes additive and dominance main effects of 1,619 markers, all additive × additive interactions, additive × dominance interactions, dominance × additive interactions, and dominance × dominance interactions, with a total of more than 5 million possible effects. The quantitative traits considered in this study include yield per plant and three yield component traits, namely the number of panicles per plant, the number of grains per panicle and grain weight. We will demonstrate that our EBlasso identifies a number of QTLs, most of which are involved in digenic interactions, and coincide with or are close to experimentally investigated genes related to yield.

## Results

Four quantitative traits including three rice yield component traits (the number of panicles per plant, the number of grains per panicle and grain weight) and overall yield per plant were analyzed using the EBlasso method. The full QTL model includes main additive and dominance effects of 1,619 markers and all their pair-wise interactions, with a total of *k* = 5,242,322 variables (see the Materials and Methods section for the genetic map). To understand the performance gain of the full model, we also performed QTL mapping for the four traits with a QTL model including *k* = 3,238 main effects, which is referred to as the main effect model.

We estimated the phenotypic variance explained by a particular QTL *j* as 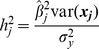, *j* = 1, 2, …, 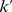, where var(***x*** *_j_*) is the variance of the coefficient of QTL *j* and the total phenotypic variance 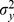 was estimated from the data. To estimate the total variance explained by all identified QTLs, we refitted the data to an ordinary linear regression model that includes variables corresponding to the identified QTLs. The phenotypic values were predicted from the linear regression model as 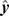, and the total phenotypic variance explained by all identified QTLs was calculated as
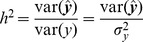
(1).

### QTL mapping for the number of panicles per plant

**Figure 1:**
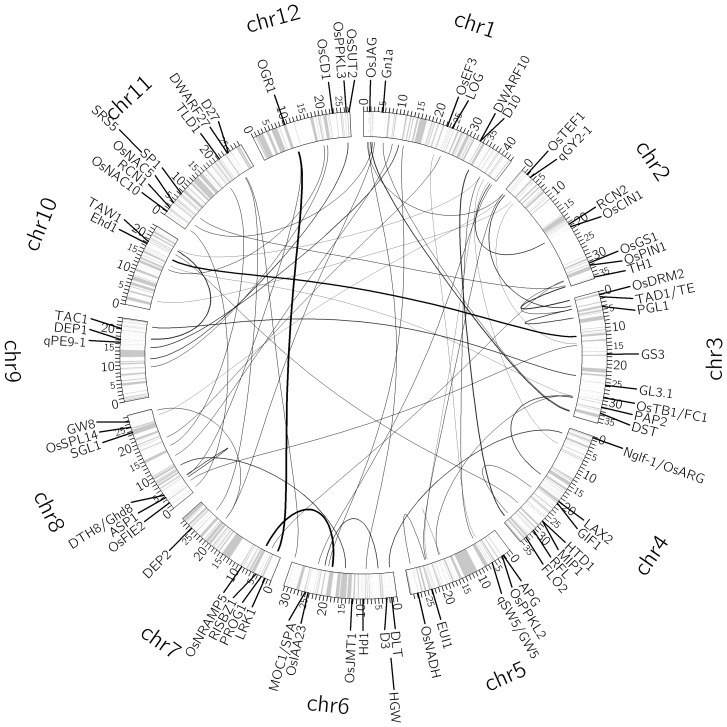
Interaction network of 103 QTLs for the number of panicles per plant. The circle shows the bin map and columns indicate position of the makers (ticks in million base pairs). The thickness of a link is proportional to the strength of the interaction effect. A short straight line indicates a main effect. Molecularly characterized genes related to yield are also labeled in the appropriate positions of the genome.

The three-step cross validation (CV) procedure (detailed in the Materials and Methods section) for the full model identified the optimal pair of parameters as (*a*, *b*) = (0.5, 0.5) (Table S1 in File S1). Using the optimal values of (*a*, *b*), the EBlasso algorithm shrunk most of *k* variables to zero and yielded a QTL model with 111 nonzero effects. The statistical test, described in the Materials and Methods section, for each nonzero effect identified 54 significant effects at a *p*-value ≤0.01 (Table 1). Among them, one was main additive effect, 18 were additive × additive interaction, 32 were additive ×dominance interaction, and three were dominance × dominance interaction. The 54 effects involved 103 QTLs and explained 94.05% of the total phenotypic variance. We did a literature survey and found 99 genes with known genomic locations that had experimental evidence showing that they were related to rice yield and yield component traits. For each of the 103 QTLs, we identified genes from 99 experimentally investigated genes that were within 20 centi-Morgan (cM) distance and associated such genes with the QTL. In total, we found 58 genes for 103 QTLs. For the ease of presentation, we organized QTLs within 20 cM distance into a group, which resulted in 51 groups for 103 QTLs. These 51 QTL groups and associated genes are listed in Table S2 in File S1. It is seen that 36 groups of QTLs have at least one associated gene and the distances between QTLs and their associated genes are relatively small (median distance 5.37 cM). Moreover, 21 QTLs involved in 19 of 54 effects locate within 2 cM distance to at least one gene influencing rice yield. The interaction network of the 103 QTLs and their associated genes are visualized in Figure 1.

**Table 1:** Estimated QTL effects from the full model for the number of panicles per plant.

Loci(*i*, *j*)^a^	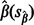 b	*p*-value^c^	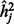 d	Loci(*i*, *j*)	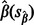	*p*-value	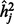
(757__add_, 757__add_)	−0.12(0.04)	2.16×10^−3^	0.0030	(104__dom_, 732__add_)	−0.11(0.05)	9.54×10^−3^	0.0012
7__add_, 220__dom_)	0.20(0.06)	2.39×10^−4^	0.0032	(186__dom_, 735__add_)	−0.20(0.06)	1.72×10^−4^	0.0039
(10__add_, 887__dom_)	−0.25(0.05)	3.78×10^−6^	0.0061	(518__dom_, 759__add_)	−0.27(0.06)	1.64×10^−6^	0.0075
(18__add_, 1407__dom_)	0.28(0.06)	1.45×10^−6^	0.0080	(220__dom_, 784__add_)	0.23(0.06)	2.38×10^−5^	0.0045
(20__add_, 1026__dom_)	0.27(0.06)	1.82×10^−6^	0.0060	(561__dom_, 828__add_)	−0.36(0.05)	4.88×10^−12^	0.0140
(44__add_, 532__dom_)	−0.27(0.05)	1.16×10^−7^	0.0071	(861__add_, 918__add_)	0.41(0.05)	1.11×10^−15^	0.0182
(69__add_, 913__dom_)	0.21(0.05)	2.39×10^−5^	0.0040	(904__add_, 1113__dom_)	0.33(0.05)	3.50×10^−10^	0.0098
(123__add_, 1132__add_)	0.23(0.05)	7.78×10^−7^	0.0053	(213__dom_, 929__add_)	0.27(0.05)	4.85×10^−8^	0.0076
(166__add_, (684__add_)	0.46(0.04)	<10^−15^	0.0279	(967__add_, 1515__add_)	0.20(0.05)	2.91×10^−5^	0.0040
(186__add_, 1372__add_)	−0.11(0.04)	2.86×10^−3^	0.0013	(908__dom_, 994__add_)	0.90(0.05)	<10^−15^	0.0782
(192__add_, 580__add_)	−0.11(0.04)	8.35×10^−3^	0.0013	(1026__add_, 1173__add_)	0.21(0.05)	9.05×10^−6^	0.0044
(199__add_, 782__dom_)	−0.08(0.03)	2.53×10^−3^	0.0006	(1037__add_, 1510__add_)	0.14(0.05)	1.13×10^−3^	0.0018
(208__add_, 309__add_)	−0.31(0.05)	1.50×10^−9^	0.0092	(1089__dom_, 1096__add_)	0.34(0.12)	2.17×10^−3^	0.0016
(227__add_, 364__dom_)	−0.36(0.05)	3.18×10^−12^	0.0145	(1119__add_, 1471__add_)	−0.19(0.04)	1.29×10^−5^	0.0045
(244__add_, 1303__dom_)	−0.11(0.04)	5.10×10^−3^	0.0012	(229__dom_, 1160__add_)	−0.11(0.04)	5.70×10^−3^	0.0012
(249__add_, 417__dom_)	0.12(0.04)	3.14×10^−3^	0.0015	(1208__add_, 1583__dom_)	−0.14(0.05)	3.20×10^−3^	0.0015
(333__add_, 991__add_)	0.24(0.05)	3.92×10^−7^	0.0057	(64__dom_, 1223__add_)	−0.43(0.05)	2.22×10^−15^	0.0191
(335__add_, 372__add_)	0.20(0.05)	2.05×10^−5^	0.0041	(1237__add_, 1370__add_)	0.54(0.05)	<10^−15^	0.0279
(349__add_, 1425__dom_)	−0.23(0.05)	3.20×10^−6^	0.0060	(1334__add_, 1576__add_)	0.22(0.05)	2.94×10^−6^	0.0049
(354__add_, 358__dom_)	−0.50(0.05)	<10^−15^	0.0233	(408__dom_, 1356__add_)	−0.73(0.05)	<10^−15^	0.0488
(371__dom_, 381__add_)	0.37(0.05)	6.01×10^−11^	0.0113	(1065__dom_, 1394__add_)	−0.17(0.05)	1.55×10^−4^	0.0026
(421__add_, 1079__add_)	−0.15(0.04)	1.04×10^−4^	0.0023	(981__dom_, 1558__add_)	−0.79(0.05)	<10^−15^	0.0735
(456__add_, 1282__add_)	0.38(0.05)	9.99×10^−15^	0.0167	(1094__dom_, 1558__add_)	0.21(0.05)	4.05×10^−5^	0.0046
(517__add_, 1346__add_)	−0.10(0.04)	8.46×10^−3^	0.0009	(1217__dom_,1615__add_)	0.37(0.05)	5.88×10^−13^	0.0144
(520__add_,595__dom_)	−0.37(0.05)	4.03×10^−11^	0.0109	(54__dom_, 1117__dom_)	0.27(0.05)	3.96×10^−7^	0.0052
(15__dom_, 534__add_)	0.47(0.05)	<10^−15^	0.0246	(627__dom_, 681__dom_)	−0.25(0.05)	1.05×10^−6^	0.0048
(649__add_, 1364__add_)	−0.20(0.04)	3.80×10^−6^	0.0045	(786__dom_, 810__dom_)	0.15(0.05)	1.11×10^−3^	0.0021
Parameter(s)				*a* = 0.5, *b* = 0.5			
*μ*				0.0035			
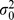				0.1444			
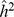				0.9405			

*^a^*add: additive effect; dom: dominance effect. If *i* equals *j*, then it is a main effect, otherwise, it is an interaction between locus *i* and locus *j*. Total number of effects is 112, only 54 effects with a *p*-value ≤0.01 are listed in this table.

*^b^*The estimated marker effect is denoted by 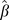 and the standard deviation is denoted by 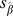.

*^c^* *P*-value is obtained via *t*-test.

*^d^*Phenotypic variation explained.

The three-step CV for the main effect model identified the optimal pair of parameters as (*a*, *b*) = ( −0.01, 0.5) (Table S1 in File S1), with which eight additive and two dominance effects involving ten QTLs were identified with a *p*-value ≤0.01 (Table 2). The ten effects totally explained 39.76% of the phenotypic variance, and nine of them had genes related to yield within 20 cM distance (median distance 9.29 cM) (Table S3 in File S1). Seven QTLs were identical to QTLs or within the QTL group identified from the full model (Bins 228, 353, 757, 861, 908, 994, 1363), and the other three (Bins 3, 461 and 818) were close to QTLs identified by the full model. Specifically, Bin3 was 3.97 cM away from Bin7 identified from the full model; Bin461 was 8.29 cM away from Bin456 identified from the full model; and Bin818 was 6.15 cM away from Bin810 identified from the full model. Comparing the results obtained from the two models, we see that the full model identified more QTLs, which included all those identified by the main effect model, and explained a much larger percentage of the phenotypic variance.

**Table 2:** Estimated QTL effects from the main effect model for the number of panicles per plant.

locus^a^	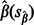 b	*p*-value^c^	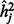 d
3__add_	0.22(0.09)	6.86×10^−3^	0.0088
228__add_	−0.24(0.09)	2.98×10^−3^	0.0125
353__add_	−0.24(0.09)	2.68×10^−3^	0.0126
757__add_	−0.54(0.10)	4.18×10^−8^	0.0625
818__add_	0.40(0.10)	4.82×10^−5^	0.0300
908__add_	0.31(0.09)	4.78×10^−4^	0.0206
994__add_	0.54(0.11)	3.53×10^−7^	0.0524
1363__add_	−0.26(0.09)	2.29×10^−3^	0.0135
461__dom_	0.30(0.10)	1.92×10^−3^	0.0091
861__dom_	−0.45(0.10)	1.17×10^−5^	0.0209
Parameter(s)		*a* = −0.01, *b* = 0.5	
*μ*		−0.0600	
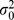		1.5260	
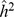		0.3976	

*^a^*add: additive effect; dom: dominance effect. Total number of effects is 10, all with a *p*-value ≤0.01.

*^b^*The estimated marker effect is denoted by 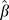 and the standard deviation is denoted by 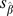.

*^c^* *P*-value is obtained via *t*-test.

*^d^*Phenotypic variation explained.

### QTL mapping for the number of grains per panicle

**Figure 2:**
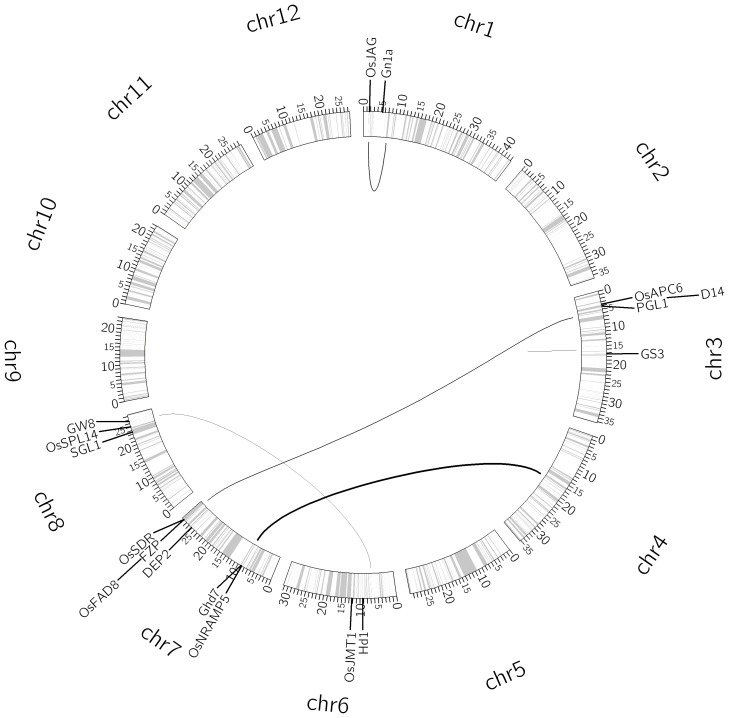
Interaction network of nine QTLs for the number of grains per panicle. The circle shows the bin map and columns indicate position of the makers (ticks in million base pairs). The thickness of a link is proportional to the strength of the interaction effect. A short straight line indicates a main effect. Molecularly characterized genes related to yield are also labeled in the appropriate positions of the genome.

The CV analysis identified the optimal pair of parameters (*a*, *b*) = (0.05, 0.1) for the full QTL model for the number of grains per panicle (Table S4 in File S1), with which EBlasso identified five nonzero effects. All of these nonzero effects were significant at a *p*-value ≤0.01 (Table 3), including one main additive effect and four additive × dominance interactions. The five effects involved nine QTLs, and explained 46.51% of the overall phenotypic variance. Eight of the nine QTLs have experimentally verified genes related to rice yield within 20 cM distance (median distance 4.86 cM) (Table S5 in File S1). Moreover, four of these QTLs involved in four effects locate within 2 cM distance to at least one yield-related gene. The interaction network of the nine QTLs and their associated genes are depicted in Figure 2.

**Table 3:** Estimated QTL effects from the full model for the number of grains per panicle.

Loci(*i*, *j*)*^a^*	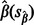 *^b^*	*p*-value*^c^*	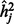 *^d^*
(436__add_, 436__add_)	6.79(0.98)	1.58×10^−11^	0.0846
(10__dom_, 50__add_)	−8.74(1.41)	1.05×10^−9^	0.0695
(875__add_, 1156__dom_)	7.15(1.44)	6.37×10^−7^	0.0427
(595__dom_, 1004__add_)	−12.78(1.88)	3.34×10^−11^	0.0853
(381__dom_, 1057__add_)	−8.82(1.33)	9.40×10^−11^	0.0801
Parameter(s)		*a* = 0.05, *b* = 0.1	
*μ*		−0.3228	
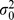		156.7998	
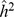		0.4651	

*^a^*add: additive effect; dom: dominance effect. If *i* equals *j*, then it is a main effect, otherwise, it is an interaction between locus *i* and locus *j*. Total number of effects is 5, all with a *p*-value ≤0.01.

*^b^*The estimated marker effect is denoted by 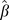 and the standard deviation is denoted by 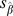.

*^c^* *P*-value is obtained via *t*-test.

*^d^*Phenotypic variation explained.

The same three-step CV for the main effect model identified the optimal pair of parameters (*a*, *b*) = ( −0.4, 0.5) (Table S4 in File S1), with which five additive effects were identified, all having a *p*-value ≤0.01 (Table 4). The five QTLs (Bins 43, 436, 877, 1006, 1057) totally explained 41.48% of the phenotypic variance, and all had molecularly characterized genes related to rice yield within 19 cM distance (median distance 1.59 cM) (Table S6 in File S1). All five QTLs were identical or very close to the QTLs identified from the full model. Specifically, Bin436 and Bin1057 were identified in both models; Bin43 is 3.40 cM away from Bin50 identified from the full model; Bin877 is 0.47 cM away from Bin875 identified from the full model; and Bin1006 is 0.72 cM away from Bin1004 identified from the full model. Comparing the results obtained from the two models, we observed that although both models identified five effects, the full model identified four more QTLs and explained a slightly larger percentage of phenotypic variance. Moreover, the main effect model identified five additive effects, but the full model identified QTLs with both additive and dominance effects.

**Table 4:** Estimated QTL effects from the main effect model for the number of grains per panicle.

locus^a^	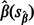 b	*p*-value^c^	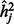 d
43__add_	−5.15(1.08)	1.38×10^−6^	0.0454
436__add_	8.06(1.03)	6.02×10^−14^	0.1190
877__add_	3.64(1.05)	3.21×10^−4^	0.0225
1006__add_	−8.00(1.15)	1.48×10^−11^	0.1036
1057__add_	−7.51(1.11)	3.55×10^−11^	0.0973
Parameter(s)		*a* = −0.4, *b* = 0.5	
*μ*		−0.6700	
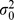		171.21	
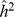		0.4148	

*^a^*add: additive effect; dom: dominance effect. Total number of effects is five, all with a *p*-value ≤0.01.

*^b^*The estimated marker effect is denoted by 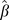 and the standard deviation is denoted by 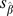.

*^c^* *P*-value is obtained via *t*-test.

*^d^*Phenotypic variation explained.

### QTL mapping for grain weight

**Figure 3:**
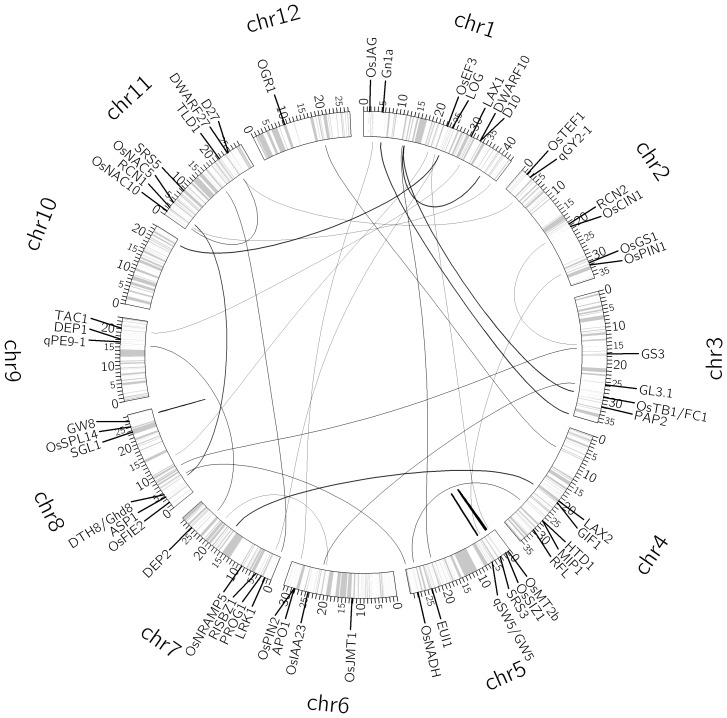
Interaction network of 52 QTLs for grain weight. The circle shows the bin map and columns indicate position of the makers (ticks in million base pairs). The thickness of a link is proportional to the strength of the interaction effect. A short straight line indicates a main effect. Molecularly characterized genes related to yield are also labeled in the appropriate positions of the genome.

The CV analysis determined the optimal (*a*, *b*) = (1, 1) (Table S7 in File S1) for the full QTL model for grain weights. Using the optimal *a* and *b*, EBlasso yields a QTL model including 89 nonzero effects, among which 28 effects were identified as significant at a *p*-value ≤0.01 (Table 5). Among them, one was a main additive effect, 10 were additive × additive, 15 were additive ×dominance, and two were dominance × dominance interactions. The 28 effects involved 52 QTLs, and explained 93.79% of the phenotypic variance. QTLs with a distance ≤20 cM were placed into a group, resulting in 32 groups, and 26 of the 32 QTL groups had at least one gene within 20 cM distance (median distance 5.06 cM) (Table S8 in File S1). Moreover, 15 QTLs involved in 12 of 28 effects locate within 2 cM distance to at least one yield-related gene. The interaction network of the 52 QTLs and their associated genes are shown in Figure 3.

**Table 5:** Estimated QTL effects from the full model for grain weight.

Loci(*i*, *j*)^a^	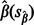 b	*p*-value^c^	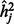 d
(729__add_, 729__add_)	1.02(0.07)	<10^−15^	0.1548
(37__add_, 547__dom_)	0.71(0.09)	1.29×10^−14^	0.0428
(67__add_, 772__add_)	−0.25(0.08)	6.56×10^−4^	0.0047
(96__add_, 1117__dom_)	0.21(0.08)	2.95×10^−3^	0.0035
(119__add_, 987__add_)	0.18(0.07)	6.73×10^−3^	0.0024
(151__add_, 1262__add_)	−0.15(0.07)	9.79×10^−3^	0.0018
(71__dom_, 184__add_)	−0.67(0.09)	1.55×10^−13^	0.0374
(210__add_, 1400__add_)	0.19(0.08)	7.79×10^−3^	0.0025
(329__add_, 727__dom_)	0.22(0.09)	4.63×10^−3^	0.0040
(310__dom_, 419__add_)	−0.21(0.05)	1.86×10^−5^	0.0043
(431__add_, 1111__add_)	0.35(0.08)	1.01×10^−5^	0.0107
(71__dom_, 500__add_)	−0.76(0.08)	<10^−15^	0.0493
(583__add_, 1578__dom_)	0.35(0.08)	9.50×10^−6^	0.0092
(107__dom_, 700__add_)	0.19(0.07)	4.80×10^−3^	0.0035
(708__dom_, 714__add_)	−1.15(0.32)	1.79×10^−4^	0.0076
(818__add_, 1100__add_)	0.26(0.08)	3.93×10^−4^	0.0053
(916__add_, 1026__add_)	0.15(0.06)	8.50×10^−3^	0.0019
(472__dom_, 920__add_)	−0.27(0.09)	1.65×10^−3^	0.0058
(18__dom_, 955__add_)	−0.20(0.08)	3.77×10^−3^	0.0033
(971__add_, 1461__add_)	0.27(0.08)	4.75×10^−4^	0.0075
(620__dom_, 1011__add_)	−0.67(0.09)	3.31×10^−12^	0.0336
(1035__add_, 1224__add_)	0.30(0.07)	3.27×10^−5^	0.0081
(1093__add_, 1407__dom_)	0.44(0.08)	2.13×10^−7^	0.0148
(1167__dom_, 1168__add_)	−0.47(0.16)	1.37×10^−3^	0.0051
(119__dom_, 1375__add_)	0.61(0.09)	7.83×10^−11^	0.0289
(1397__add_, 1505__add_)	0.41(0.09)	1.95×10^−6^	0.0119
(247__dom_, 1505__dom_)	−0.23(0.08)	1.22×10^−3^	0.0032
(647__dom_, 796__dom_)	0.26(0.08)	4.29×10^−4^	0.0044
Parameter(s)		*a* = 1, *b* = 1	
*μ*		−0.0661	
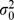		0.5317	
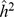		0.9379	

*^a^*add: additive effect; dom: dominance effect. If *i* equals *j*, then it is a main effect, otherwise, it is an interaction between locus *i* and locus *j*. Total number of effects is 90, only 28 effects with a *p*-value ≤0.01 are listed in this table.

*^b^*The estimated marker effect is denoted by 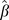 and the standard deviation is denoted by 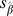.

*^c^* *P*-value is obtained via *t*-test.

*^d^*Phenotypic variation explained.

The CV analysis for the main effect model identified the optimal pair of parameters (*a*, *b*) = (1, 1) (Table S7 in File S1), with which 26 QTLs (19 additive and 7 dominance effects) were identified with a *p*-value ≤0.01 (Table 6). The 26 QTLs totally explained 84.24% of the overall phenotypic variance, and 23 of them had molecularly characterized genes related to rice yield within 16 cM distance (median distance 4.08 cM) (Table S9 in File S1). Twenty three of the 26 QTLs were identical to or within a QTL group identified from the full model, but three QTLs (Bins 228, 843, and 894) do not correspond to any QTLs identified from the full model within 20 cM distance. Again, the full model identified more QTLs than the main effect model and the QTLs detected by the full model explained more phenotypic variance than those detected by the main effect model.

**Table 6:** Estimated QTL effects from the main effect model for grain weight.

locus^a^	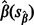 b	*p*-value^c^	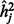 d
37__add_	0.40(0.08)	8.22×10^−7^	0.0262
50__add_	0.21(0.08)	3.14×10^−3^	0.0072
151__add_	−0.18(0.07)	2.77×10^−3^	0.0052
173__add_	−0.49(0.07)	6.46×10^−11^	0.0418
199__add_	0.23(0.06)	1.51×10^−4^	0.0077
332__add_	0.30(0.06)	1.26×10^−6^	0.0150
440__add_	−0.98(0.06)	<10^−15^	0.1670
498__add_	−0.35(0.06)	5.18×10^−8^	0.0188
710__add_	0.17(0.06)	2.13×10^−3^	0.0047
729__add_	0.81(0.07)	<10^−15^	0.0968
894__add_	−0.18(0.06)	8.21×10^−4^	0.0053
936__add_	−0.44(0.07)	3.11×10^−10^	0.0291
1008__add_	−0.34(0.06)	6.63×10^−8^	0.0177
1110__add_	0.18(0.05)	4.85×10^−4^	0.0055
1176__add_	−0.26(0.06)	8.77×10^−6^	0.0108
1251__add_	0.37(0.07)	4.64×10^−8^	0.0171
1374__add_	0.33(0.06)	2.55×10^−7^	0.0167
1442__add_	−0.22(0.06)	6.37×10^−5^	0.0079
1565__add_	0.20(0.06)	2.33×10^−4^	0.0063
38__dom_	0.29(0.07)	3.18×10^−5^	0.0066
228__dom_	−0.20(0.07)	1.60×10^−3^	0.0032
312__dom_	0.17(0.06)	3.20×10^−3^	0.0024
441__dom_	−0.23(0.07)	7.95×10^−4^	0.0043
547__dom_	0.15(0.06)	6.68×10^−3^	0.0019
843__dom_	0.16(0.06)	5.41×10^−3^	0.0021
1506__dom_	−0.26(0.07)	1.89×10^−4^	0.0054
Parameter(s)		*a* = 1, *b* = 1	
*μ*		0.1000	
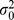		0.5224	
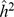		0.8424	

*^a^*add: additive effect; dom: dominance effect. Total number of effects is 38, only 30 effects with a *p*-value ≤0.01 are listed in this table.

*^b^*The estimated marker effect is denoted by 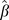 and the standard deviation is denoted by 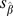.

*^c^* *P*-value is obtained via *t*-test.

*^d^*Phenotypic variation explained.

### QTL mapping for yield per plant

**Figure 4:**
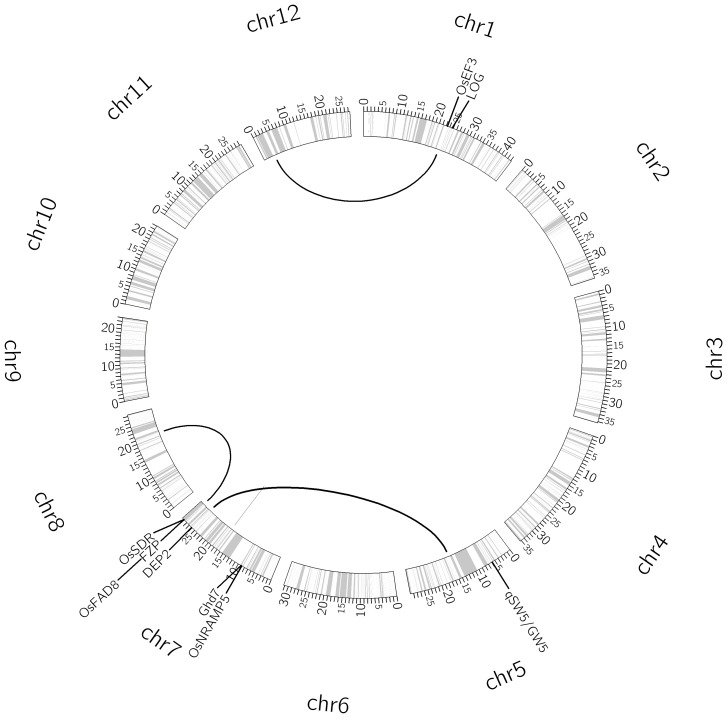
Interaction network of seven QTLs for yield per plant. The circle shows the bin map and columns indicate position of the makers (ticks in million base pairs). The thickness of a link is proportional to the strength of the interaction effect. A short straight line indicates a main effect. Molecularly characterized genes related to yield are also labeled in the appropriate positions of the genome.

The CV analysis determined the optimal pair of parameters (*a*, *b*) = (1, 1) for the full QTL model for rice yield (Table S10 in File S1). Using the optimal values of (*a*, *b*), EBlasso yielded four nonzero effects, all were significant at a *p*-value ≤0.01: one main additive effect, one additive × additive interaction, one additive ×dominance interaction, and one dominance × dominance interaction (see Table 7). The four effects involved seven QTLs and explained 34.01% of the overall phenotypic variance. Five out of the seven QTLs have an experimentally verified gene within 15 cM distance (median distance 2.21 cM) (Table S11 in File S1). Moreover, two QTLs involved in two of four effects locate within 2 cM distance to at least one yield-related gene. The interaction network of the seven QTLs and their associated genes are described in Figure 4.

**Table 7:** Estimated QTL effects from the full model for yield per plant.

Loci(*i*, *j*)^a^	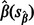 b	*p*-value^c^	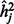 d
(1014__add_,1014__add_)	−1.94(0.37)	1.95×10^−7^	0.0544
(113__add_, 1547__add_)	−2.81(0.53)	1.43×10^−7^	0.0552
(1057__add_,1144__dom_)	−2.89(0.53)	5.57×10^−8^	0.0608
(743__dom_,1043__dom_)	3.21(0.52)	8.38×10^−10^	0.0598
Parameter(s)		*a* = 1, *b* = 1	
*μ*		−0.7521	
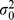		22.9734	
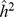		0.3401	

*^a^*add: additive effect; dom: dominance effect. If *i* equals *j*, then it is a main effect, otherwise, it is an interaction between locus *i* and locus *j*. Total number of effects is 4, all with a *p*-value ≤0.01.

*^b^*The estimated marker effect is denoted by 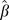 and the standard deviation is denoted by 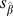.

*^c^* *P*-value is obtained via *t*-test.

*^d^*Phenotypic variation explained.

The optimal pair of parameters determined by the CV analysis for the main effect model was (*a*, *b*) = (−0.5, 0.1) (Table S10 in File S1), with which four QTLs with a *p*-value ≤0.01 were identified (Table 8). The four QTL effects explained 23.79% of the phenotypic variance, and all had at least one gene within 17 cM distance (median distance 7.82 cM) (Table S12 in File S1). Two of the four QTLs (Bin1014, Bin1057) were identical to the QTLs identified from the full model, but the other two QTLs do not correspond to any QTL identified from the full model within 20 cM distance. Overall, although the full model did not detect all QTLs identified by the main effect model, it still detected more QTLs and explained more phenotypic variance.

**Table 8:** Estimated QTL effects from the main effect model for yield per plant.

locus^a^	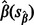 b	*p*-value^c^	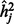 d
181__add_	−1.87(0.42)	6.24×10^−6^	0.0518
1014__add_	−2.27(0.43)	9.58×10^−8^	0.0743
1057__add_	−2.41(0.43)	2.58×10^−8^	0.0848
1100__add_	1.17(0.39)	1.35×10^−3^	0.0205
Parameter(s)		*a* = −0.5, *b* = 0.1	
*μ*		−0.1500	
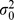		26.2580	
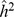		0.2379	

*^a^*add: additive effect; dom: dominance effect. Total number of effects is 4, all with a *p*-value ≤0.01.

*^b^*The estimated marker effect is denoted by 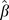 and the standard deviation is denoted by 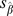.

*^c^* *P*-value is obtained via *t*-test.

*^d^*Phenotypic variation explained.

### Effect types and pleiotropic genes

Among the five types of effects (main additive, main dominance effects, additive × additive, additive ×dominance, and dominance × dominance interactions) considered in the EBlasso full models for four traits, no main dominance effects was detected, but several dominance × dominance interactions (one for rice yield, three for the number of panicles per plant, and two for grain weight) were identified. Many additive ×dominance interaction effects were identified, including one for rice yield, 32 for the number of panicles per plant, four for the number of grains per panicle, and 15 for grain weight. Phenotypic variance explained by a single effect is relatively small for all traits (Tables 1, 3, 5 and 7). For example, the largest effect has 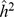  = 7.82% (908__dominance_×994__additive_) for the number of panicles per plant, 8.53% (595__dominance_×1004__additive_) for the number of grains per panicle, 15.48% (729__additive_) for grain weight, and 6.08% (1057__additive_×1144__dominance_) for yield per plant. Each main effect detected by the main effect model also explained a small percentage of the total phenotypic variance.

Many molecularly characterized genes related to yield are known to play pleiotropic roles in regulating grain productivity [31]. Without surprise, a number of such genes coincide with or close to the QTLs that were identified by our EBlasso for multiple traits, although they did not necessarily have pleiotropic effects. For example, gene *Ghd7*, *OsNRAMP5* and *DEP2* are close to several QTLs common for the four phenotypes, *qSW5/GW5*, *OsEF3* and *LOG* are near the QTLs for three phenotypes except the number of grains per panicle, and *Gn1a*, *OsJAG*, *GS3*, *OsJMT1*, *OsSPL14*, *GW8/OsSPL16*, *SGL1* are associated with QTLs for three phenotypes except yield per plant. Besides *Ghd7*, *OsNRAMP5* and *DEP2*, gene *FZP*, *OsSDR*, and *OsFAD8* was near QTLs for both yield per plant and the number of grains per panicle; 14 genes were close to QTLs for both the number of panicles per plant and the number of grains per panicle; and 62 other genes were associated with QTLs for both the number of grains per panicle and grain weight. While the pleiotropic effect of some genes have been reported [32], our QTL mapping results identified a number of genes associated with multiple phenotypes, implying their possible pleiotropic role worthy of further experimental investigation. Moreover, it is also possible that the QTLs we detected may be closely linked to unknown genes, which, if identified, will yield more insight into the molecular basis of phenotypes [1].

## Discussion

Due to its small genome and close relatedness with other grass crops, rice has served as a model plant for investigating genetic factors underlying crop productivity [33], [34]. To date, more than 600 rice genes have been experimentally cloned with related traits including yield, biotic and abiotic stresses, grain quality, plant architecture, fertility, etc. [2]. However, there is still a knowledge gap regarding the molecular basis of yield-related biological processes [1], suggesting the importance of systematic tools that can enable to understand functional role of genes [2], [35]. In this study, we employed a multiple QTL model that included all additive and dominance main effects of 1,619 markers, and all their pair-wise interactions with a total of more than 5 million possible effects, and then applied our EBlasso algorithm to identify QTLs for four agronomic related traits of rice, including yield, the number of panicles per plant, the number of grains per panicle and grain weight. Our QTL mapping revealed a number of QTLs for four traits, most of which are involved in digenic interactions. Moreover, most of these QTLs have at least one experimentally cloned gene within 20 cM distance.

The same set of markers in the recombinant inbred line (RIL) population where the “immortalized F_2_” (IMF_2_) was derived from were used for QTL mapping, via a composite interval mapping method with a scan window size of five markers [12]. Upon development of the IMF_2_ population, this dataset was obtained and the ANOVA method was applied to each pair of markers to identify both main and digenic interaction effects from 5,242,322 possible effects [24]. The composite interval mapping identified zero, three (Bin40, Bin446 and Bin1006), seven (Bins 49, 171, 439, 729, 928, 1008, and 1266), and one (Bin1007) QTLs for the number of panicles per plant, the number of grains per panicle, grain weight and yield per plant, respectively. The ANOVA method detected thousands (1432, 2696, 3524 and 2251) of digenic interactions between two bins with a *p*-value ≤0.001; and after those digenic interactions involving adjacent bins were merged, 115, 189, 238, and 204 effects were reported, respectively [24]. In contrast, our EBlasso method identified a reasonable number of effects and QTLs for each trait, and 35%–80% of identified effects for four traits involve at least one QTL that locates within 2 cM distance to at least one gene related to crop yield, which corroborates the reliability of the identified effects.

The list of genes associated with the identified QTLs provides insight into rice yield with respect to yield component traits. First, the number of panicles depends on plant's ability of producing tillers, which is under genetic, developmental and environmental influence. While previous composite interval mapping did not identify any significant effect with the same set of markers in an RIL population [12], we have identified a set of QTLs that have nearby genes known to regulate plant tillering. For example, among genes in Table S2 in File S1, *MOC1/SPA* is the first gene characterized for rice tillering; it initiates axillary buds that grow into lateral braches [36]. *OsTB1/FC1* has been identified as an important gene that negatively regulates lateral branching in rice [37]. *OsSPL14* is a highly expressed gene in the shoot apex and primordial of primary and secondary branches, which promotes panicle branching while reducing tiller number [38]. Through gene mutations, *D3*, *D10, D14*, *D17/HTD1*, and *D27* were found to affect tiller initiation and/or outgrowth [37]. Secondly, the number of grains per panicle is another important trait determining crop yield. While composite interval mapping identified three QTLs (Bin40, Bin446 and Bin1006) close to genes *Gn1a*, *GS3*, *OsNRAMP5* and *Ghd7*, our EBlasso also identified these genes in addition to other 13 genes. Among them, *FZP* is known to control spikelet meristem identity [39], *Ghd7* is a pleiotropic gene affecting grain number, plant height and heading date [40], *GW8/OsSPL16, PGL*, and *DEP2* all are known to be essential in regulating cell proliferation or elongation [41]–[43]. Thirdly, composite interval mapping detected seven QTLs (Bins 49, 171, 439, 729, 928, 1008, and 1266) for grain weight, with nearby genes *Gn1a*, *LAX1*, *GS3*, *GS5*, *qSW5/GW5*, *OsJMT1*, *OsIAA23*, *Ghd7*, *OsNRAMP5*, *TAC1*, *LGD1* and *SG1*. In addition to these genes, our EBlasso identified many other genes with known effects in controlling grain weight. For example, *GIF1* is a gene encoding a cell-wall invertase required for carbon partitioning during early grain filling, and overexpression of *GIF1* leads to larger and heavier grain weight [44]. Genes *SRS3* and *SRS5* have been found to regulate seed cell elongation [45], [46]. Over-expression of *LRK1* gene results in enhanced cellular proliferation and increased grain weight [47]. Finally, yield per plant is the most complex trait and a small number of effects were identified compared with its component traits. While composite interval mapping identified only one QTL (Bin1007) with nearby gene *Ghd7* and *OsNRAMP5*, our EBlasso identified this QTL and six other QTLs, four of which have cloned gene within 15 cM distance (Table S11 in File S1).

In conclusion, taking advantage of the powerful EBlasso model for simultaneously accounting for more than 5 million possible effects, we identified a number of QTLs for four traits of the elite rice hybrid Shanyou 63, a vast majority of which are involved in digenic interactions. This set of QTLs not only shed light on the genetic basis of the yield of the rice hybrid, but also provide candidate loci for identification of new genes that may be involved in crop yield.

## Materials and Methods

### Plant materials and QTLs

The genotype and phenotype data used in this study were obtained from previous studies [12], [24]. The mapping plants were created by first crossing between *indica* rice Zhensha 97 and Minghui 63 [7] to produce the elite rice hybrid Shanyou 63 that was the most widely cultivated in China in 1980s –1990s [24]. Then a population of 240 F_9_ RILs was derived from single-seed descent of Shanyou 63. Next, an “immortalized F_2_” (IMF_2_) population consisted of 278 crosses was created by intercrossing RILs for QTL mapping study [7], [23]. The crossed population was field tested on the experimental farm of Huazhong Agricultural University in Wuhan, China, in 1999, for traits including yield per plant, the number of panicles per plant, the number of grains per panicle and grain weight.

The RILs were genomic sequenced with an Illumina Genome Analyzer II using the bar-coded multiplexed sequencing approach as described in [25], and 270,820 high quality SNPs were identified. Bin maps were constructed by lumping consecutive SNPs with the same genotype into blocks, masking blocks with less than 250 kb to avoid false double recombinations, and merging recombination bins less than 5 kb, resulting in a map consisting of 1,619 bins without missing data [12]. Genotypes of the IMF_2_ crosses were deduced according to genotypes of their RIL parents [24]. The three genotypes in each bin were coded as A and B for each parental homozygote genotype and H for the heterozygote. Using the recombinant bins as QTLs, a 1,625.5 cM genetic linkage map was constructed with about 1.0 cM (230 kb) in length per bin (Figure 1).

### Bayesian Lasso linear regression model for multiple QTLs

We employed a Bayesian Lasso (BLasso) multiple linear regression model to infer genotypes and quantitative trait associations. The regression model includes main additive and dominance effects of 1,619 SNP bins and all their pair-wise interactions. Let *y_i_* be the phenotypic value of a quantitative trait of the *i*th individual in a mapping population. In this study we observed *y_i_*, *i* = 1, ···, *n*, of *n* = 278 individuals and collected them into a vector ***y*** = [*y_1_, y_2_,* ···, *y_n_*]*^T^*. In these *n* individuals, let *m = *1,619 denote the number of genetic markers genotyped whose main effects include additive and dominance effects. Let the additive and dominance genotypes of marker *j* of individual *i* be *x_Aij_* and *x_Dij_*, respectively, where *x_Aij_* takes on values +1, 0 and −1, and *x_Dij_* takes on values 0, +1 and 0, corresponding to genotypes A, H and B, respectively. Let us define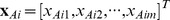 and 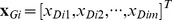. The interactions between any two effects are modeled as element-wise product of the corresponding main effects. Let **x** *_AAi_*, **x** *_ADi_*, **x** *_DAi_*, and **x** *_DDi_* be 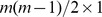 vectors containing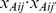, 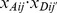,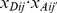, and 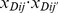, respectively, where 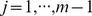 and 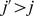. Then we have the following linear regression model for ***y***:
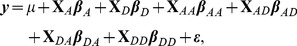
(2)where *μ* is the population mean, vectors ***β*** *_A_* and ***β*** *_D_* represent the main additive and dominance effects of all markers, respectively, and vectors ***β*** *_AA_*, ***β*** *_AD_*, ***β*** *_DA_* and ***β*** *_DD_* capture the additive × additive, additive × dominance, dominance × additive, and dominance × dominance interactions, respectively. Matrices 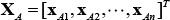, 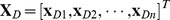, 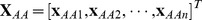, 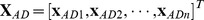, 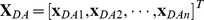, and 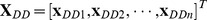
are the corresponding design matrices of different effects, and 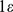 is the residual error that follows a normal distribution with zero mean and variance 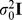.

Given *m* markers, the size of matrix **X** *_A_* or **X** *_D_* is *n*×*m*, and the size of **X** *_AA_*, **X** *_AD_*, **X** *_DA_*, or **X** *_DD_* is *n*×*q*, where *q* = *m*(*m*−1)/2 = 1,309,771. Defining 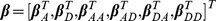, and 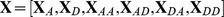, we can write (2) in a more compact form:
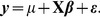
(3)


The size of matrix **X** is *n*×*k*, where *k* = 2*m*+4*q = *5,242,322, and we apparently have 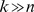. However, we would expect that most elements of 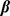 are zeros and thus we have a sparse linear model. The Blasso model employs a three-level hierarchical prior distribution to model the sparsity. At the first level, let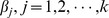, follows an independent normal distribution with mean zero and unknown variance 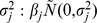. At the second level, let 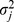, *j* = 1, 2, ···, *k*, follows an independent exponential distribution with a common parameter *λ*: 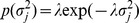. At the third level, we assign a conjugate Gamma prior *Gamma*(*a*, *b*) with a shape parameter *a* and an inverse scale parameter *b* to the parameter *λ*. Finally, we assign non-informative uniform priors to *μ* and 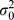. The three-level hierarchical model has two hyperparameters (*a*, *b*) for adjusting the degree of shrinkage, and cross validation (CV) can be applied to choose appropriate values of these parameters.

The QTL model (2) or equivalently (3) includes all main effects and digenic interactions. We refer to this model as the full model throughout the paper. We also performed QTL mapping with the model 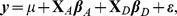 which is referred to as the main effect model, since it includes only the main effects.

### Model inference and cross validation

The Blasso model can be inferred efficiently with the empirical Blasso (EBlasso) algorithm [21]. The EBLasso algorithm employs a coordinate ascent method to find 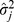, the estimate of 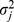, *j* = 0 …, *k*, that maximizes the likelihood function of 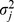, *j* = 0, …, *k*. In the iterative process, many 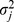 or equivalent 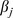 are shrunk to zero. The coordinate ascent method along with other algorithmic techniques makes the EBlasso algorithm very efficient. Our previous studies demonstrated that EBlasso outperformed several other multiple QTL mapping methods including the empirical Bayes method [26], the Bayesian hierarchical generalized linear models (BhGLM) [27], HyperLasso [28], and Lasso [29]. Detailed description of the EBlasso algorithm can be found in [21], [22] and an efficient C program with the R interface [30] implementing the EBlasso algorithm is available.

The optimal values of two hyperparameters (*a*, *b*) of the EBLasso algorithm were obtained with five-fold CV in three steps to minimize the prediction error (*PE*) calculated from 
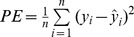
, where 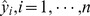, is the estimated phenotypic value. In the first step, *a* = *b* = 0.001, 0.01, 0.1, 1 were examined and a pair (*a*_1_, *b*_1_) corresponding to the smallest *PE* was obtained. In the second step, *b* was fixed at *b*_1_ and *a* was chosen from the set [−0.9, −0.8, −0.7, −0.6, −0.5, −0.4, −0.3, −0.2, −0.1, −0.01, 0.01, 0.05, 0.1, 0.5, 1], which yielded a value *a*_2_ corresponding to the smallest *PE*. In the third step, *a* = *a*_2_ was fixed and *b* varied from 0.01 to 10 with a step size of one for *b*>1 and a step size of one on the logarithmic scale for *b*<1. Note that when fixing one of the two parameters, the degree of shrinkage is a monotonic function of the other parameter [21], [22]. Therefore, in the second and third steps, the selection did not go through the full path but stopped if the current *PE* was one standard error larger than the minimum *PE* in previous steps.

### Statistical significance test

One advantage of the EBLasso algorithm relative to Lasso [29] is that it not only outputs a 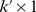 (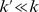) vector 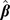
as an estimate of nonzero elements of 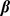, but also gives an estimate of the covariance of 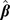, 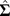. Letting 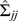 be the *j*th diagonal element of 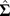, we can use the *t*-statistics 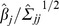 to test if 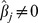 at a certain significance level.

## Supporting Information

File S1 **Tables S1–S12. Table S1.** Cross-validation for determining hyperparameters (*a*, *b*) used in QTL mapping for the number of panicles per plant. **Table S2.** Experimentally investigated genes near QTLs for the number of panicles per plant identified with the full model. **Table S3.** Experimentally investigated genes near QTLs for the number of panicles per plant identified with the main effect model. **Table S4.** Cross-validation for determining hyperparameters (*a*, *b*) used in QTL mapping for the number of grains per panicle. **Table S5.** Experimentally investigated genes near QTLs for the number of grains per panicle identified with the full model. **Table S6.** Experimentally investigated genes near QTLs for the number of grains per panicle identified with the main effect model. **Table S7.** Cross-validation for determining hyperparameters (*a*, *b*) used in QTL mapping for grain weight. **Table S8.** Experimentally investigated genes near QTLs for grain weight identified with the full model. **Table S9.** Experimentally investigated genes near QTLs for grain weight identified with the main effect model. **Table S10.** Cross-validation for determining hyperparameters (*a*, *b*) used in QTL mapping for yield per plant. **Table S11.** Experimentally investigated genes near QTLs for yield per plant identified with the full model. **Table S12.** Experimentally investigated genes near QTLs for yield per plant identified with the main effect model.
(DOC)
