# Supplementary material for: Whole-Genome Quantitative Trait Locus Mapping Reveals Major Role of Epistasis on Yield of Rice
Source: PLoS One. 2014 Jan 29;9(1):e87330. doi: 10.1371/journal.pone.0087330 (PMC3906158; doi:10.1371/journal.pone.0087330)
Supplement: File S1 — Tables S1–S12. Table S1. Cross-validation for determining hyperparameters (a, b) used in QTL mapping for the number of panicles per plant. Table S2. Experimentally investigated genes near QTLs for the number of panicles per plant identified with the full model. Table S3. Experimentally investigated genes near QTLs for the number of panicles per plant identified with the main effect model. Table S4. Cross-validation for determining hyperparameters (a, b) used in QTL mapping for the number of grains per panicle. Table S5. Experimentally investigated genes near QTLs for the number of grains per panicle identified with the full model. Table S6. Experimentally investigated genes near QTLs for the number of grains per panicle identified with the main effect model. Table S7. Cross-validation for determining hyperparameters (a, b) used in QTL mapping for grain weight. Table S8. Experimentally investigated genes near QTLs for grain weight identified with the full model. Table S9. Experimentally investigated genes near QTLs for grain weight identified with the main effect model. Table S10. Cross-validation for determining hyperparameters (a, b) used in QTL mapping for yield per plant. Table S11. Experimentally investigated genes near QTLs for yield per plant identified with the full model. Table S12. Experimentally investigated genes near QTLs for yield per plant identified with the main effect model. (DOC) [file pone.0087330.s001.doc]

# Trait 1: the number of panicles per plant

# Table S1. Cross-validation for determining hyperparameters (*a*, *b*) used in QTL mapping for the number of panicles per plant.

| **Algorithm** | **Parameters (*a*, *b*)** | ***PE* ± *STE^a^*** |
| --- | --- | --- |
|
|  | (0.01, 0.01) | 3.49 ± 0.04 |
|  | (0.1, 0.1) | 3.30 ± 0.03 |
|  | (0.5, 0.5) | 3.10 ± 0.03*^b^* |
| EBlasso | (1, 1) | 3.35 ± 0.07 |
| Full model | (0.1, 0.5) | 3.33 ± 0.04 |
|  | (0.01, 0.5) | 3.31 ± 0.03 |
|  | (-0.001, 0.5) | 3.49 ± 0.06 |
|  | (0.5, 1) | 3.27 ± 0.05 |
|  | (0.5, 2) | 3.28 ± 0.03 |
|  | (0.5, 3) | 3.43 ± 0.07 |
|  | (0.01, 0.01) | 2.35 ± 0.10 |
|  | (0.1, 0.1) | 2.28 ± 0.08 |
|  | (1, 1) | 2.28 ± 0.09 |
| EBlasso | (0.1, 0.5) | 2.28 ± 0.09 |
| Main model | (0.01, 0.5) | 2.22 ± 0.08 |
|  | (-0.01, 0.5) | 2.14 ± 0.08*^b^* |
|  | (-0.1, 0.5) | 2.31 ± 0.09 |
|  | (-0.2, 0.5) | 2.34 ± 0.10 |
|  | (-0.3, 0.5) | 2.35 ± 0.10 |
|  | (-0.01, 1) | 2.66 ± 0.11 |

*^a^*The average *PE* and standard error were obtained from five-fold cross validation.

*^b^*The optimal *PE* and its corresponding parameters used in QTL mapping.

# Table S2. Experimentally investigated genes near QTLs for the number of panicles per plant identified with the full model.

| **Bin group (chr, cM)***^a^* | **Gene/Location(chr, cM)** | **Distance (cM)***^b^* | **Reference** |
| --- | --- | --- | --- |
| 7,10,15,18,20(1,4.69-14.27) | *OsJAG*/(1, 6.15) | 0.00 |  |
| 44(1, 35.09) | *Gn1a*/(1, 30.72) | 4.37 |  |
| 54(1, 53.93) | − | − | − |
| 64,69(1, 65.38-74.48) | − | − | − |
| 104,123(1, 98.63-113.03) | *LOG*/(1, 113.27) | 0.24 |  |
|  | *OsEF3*/(1, 102.73) | 4.10 |  |
| 166,186, 192(1, 145.97-163.52) | *DWARF10*/(1, 145.25) | 0.72 |  |
|  | *D10*/(1, 145.25) | 0.72 |  |
| 199,208,213(1, 188.15-197.66) | − | − | − |
| 220,227,229(2, 3.95-8.81) | *OsTEF1*/(2, 13.87) | 5.07 |  |
|  | *qGY2-1*/(2, 17.51) | 8.70 |  |
| 244,249(2, 32.37-36.31) | − | − | − |
| 309(2, 82.78) | *RCN2*/(2, 78.18) | 4.60 |  |
|  | *OsCIN1*/(2, 82.78) | 0.00 |  |
|  | *PGL2*/(2, 149.24) | 6.91 |  |
| 333,335, 349,354(2, 156.15-174.45) | *OsPIN1*/(2, 148.00) | 8.14 |  |
|  | *OsGS1*/(2, 146.32) | 9.83 |  |
|  | *TH1*/(2, 174.45) | 0.00 |  |
|  | *OsDRM2*/(3, 1.95) | 1.71 |  |
| 358,364, 371,372(3, 0.24-20.16) | *OsAPC6*/(3, 22.66) | 2.50 |  |
|  | *TAD1*/(3, 10.68) | 9.72 |  |
|  | *PGL1*/(3, 35.14) | 14.98 |  |
| 381(3, 40.64) | *D14*/(3, 37.67) | 2.97 |  |
| 408,417,421(3, 69.50-84.18) | − | − | − |
| 456(3, 99.54) | *GS3*/(3, 94.72) | 4.82 |  |
|  | *OsPPKL1*/(3, 114.95) | 15.42 |  |
| 517,518,520(3, 141.34-142.80) | *OsTB1*/(3, 134.05) | 7.29 |  |
|  | *PAP2*/(3, 152.25) | 9.45 |  |
|  | *DST*/(3, 159.68) | 16.88 |  |
| 532,534(3, 178.71-180.67) | − | − | − |
| 561,580(4, 5.59-14.17) | *Nglf-1*/( 4, 0.48) | 5.10 |  |

*^a^* Bin ID and the map information were obtained from dataset S1 in . QTLs with a distance ≤ 20cM are represented as one group.

*^b^*Distance between the gene and the nearest QTL.

**Table S2.** continued

| **Bin group (chr, cM)***^a^* | **Gene/Location(chr, cM)** | **Distance (cM)***^b^* | **Reference** |
| --- | --- | --- | --- |
| 595(4, 27.87) | − | − | − |
| 627(4, 59.94) | *GIF1*/(4, 53.86) | 6.08 |  |
|  | *LAX2*/(4, 49.32) | 10.62 |  |
|  | *D17*/(4, 84.20) | 2.22 |  |
| 649(4, 86.42) | *MIP1*/(4, 84.2) | 2.22 |  |
|  | *RFL*/(4, 96.82) | 10.40 |  |
|  | *FLO2*/(4, 109.89) | 5.24 |  |
| 681,684(4, 106.80-115.12) | *OsAP2-39*/(4,103.18) | 3.63 |  |
|  | *OsGS2*/(4, 125.03) | 9.91 |  |
|  | *qSW5*/(5, 33.28) | 0.24 |  |
|  | *GS5*/(5, 20.52) | 13.00 |  |
| 732,735(5, 33.52-35.98) | *SRS3*/(5, 20.04) | 13.48 |  |
|  | *OsPPKL2*/(5, 17.57) | 15.96 |  |
|  | *APG*/(5, 14.58) | 18.94 |  |
| 757,759(5, 61.38-62.35) | − | − | − |
| 782,784,786(5, 80.96-85.18) | *EUI1*/(5, 90.68) | 5.49 |  |
| 810(5, 109.89) | *OsNADH*/(5, 106.49) | 3.40 |  |
|  | *DLT*/(6, 5.82) | 0.96 |  |
| 828(6, 4.86) | *D3*/(6, 12.86) | 8.00 |  |
|  | *HGW*/(6,15.77) | 10.91 |  |
| 861(6, 28.34) | − | − | − |
| 887(6, 50.98) | *Hd1*/(6, 52.42) | 1.44 |  |
|  | *MOC1*/(6, 98.749) | 11.66 |  |
| 904,908,913, 918,929(6, 68.93-87.09) | *TGW6*/(6, 102.99) | 15.90 |  |
|  | *OsIAA23*/(6, 92.88) | 5.80 |  |
|  | *OsJMT1*/(6, 61.55) | 7.39 |  |
| 967(6, 144.41) | − | − | − |
| 981(7, 7.89) | *LRK1*/(7, 8.62) | 0.73 |  |
|  | *Ghd7*/(7, 54.73) | 9.03 |  |
|  | *RISBZ1*/(7, 28.13) | 0.72 |  |
| 991,994(7, 27.41-45.70) | *WAF1*/(7, 27.41) | 0.00 |  |
|  | *PROG1*/(7, 25.67) | 1.73 |  |
|  | *OsNRAMP5*/(7, 54.25) | 8.55 |  |
|  | *OsTRXh1*/(7, 28.13) | 0.72 |  |
| 1026(7, 82.71) | − | − | − |
| 1037(7, 104.03) | *DEP2*/(7, 118.38) | 14.35 |  |

**Table S2.** continued

| **Bin group (chr, cM)***^a^* | **Gene/Location(chr, cM)** | **Distance (cM)***^b^* | **Reference** |
| --- | --- | --- | --- |
| 1065(8, 9.426) | *OsFIE2*/(8, 23.56) | 14.13 |  |
| 1079,1089, 1094,1096(8, 28.68-39.63) | *ASP1*/(8, 29.16) | 0.48 |  |
|  | *DTH8*/(8, 36.74) | 0.72 |  |
| 1113,1117, 1119,1132(8, 46.66-56.73) | − | − | − |
|  | *OsSPL14*/(8, 94.22) | 10.99 |  |
| 1160,1173(8, 105.21-118.16) | *GW8*/(8, 106.89) | 1.69 |  |
|  | *SGL1*/(8, 88.50) | 16.71 |  |
| 1208,1217(9, 13.58-21.86) | − | − | − |
|  | *DEP1*/(9, 71.77) | 2.43 |  |
| 1223,1237(9, 62.83-74.19) | *SG1*/(9, 80.28) | 6.08 |  |
|  | *qPE9-1*/(9, 65.26) | 2.44 |  |
|  | *OsEATB*/(75.65) | 1.45 |  |
| 1282(9, 94.10) | *TAC1*/(9, 86.57) | 7.52 |  |
|  | *LGD1*/(9, 86.57) | 7.52 |  |
| 1303(10, 7.00) | − | − | − |
| 1334,1346(10, 26.34-36.68) | − | − | − |
| 1356,1364, 1370,1372(10, 47.44-65.38) | *Ehd1*/(10, 49.41) | 1.97 |  |
|  | *TAW1*/(10, 51.86) | 4.42 |  |
|  | *RCN1*/(11, 17.19) | 2.73 |  |
| 1394,1407(11, 4.68-19.92) | *OsNAC5*/(11, 27.36) | 7.44 |  |
|  | *OsNAC10*/(11, 5.88) | 1.20 |  |
|  | *CycT1;3*/(11, 19.92) | 0.00 |  |
| 1425(11, 37.58) | *SP1*/(11, 46.04) | 8.46 |  |
|  | *SRS5*/(11, 52.07) | 14.49 |  |
| 1471(11, 70.46) | *TLD1*/(11, 83.27) | 12.81 |  |
| 1510,1515(11, 102.03-104.21) | *DWARF27*/(11, 100.57) | 1.46 |  |
|  | *D27*/(11, 101.79) | 0.24 |  |
| 1558,1576, 1583(12, 43.43-60.14) | *OsCD1*/(12, 77.33) | 17.19 |  |
|  | *OGR1*/(12, 42.95) | 0.48 |  |
| 1615(12, 105.07) | *OsPPKL3*/(12, 106.05) | 0.98 |  |
|  | *OsSUT2*/(12, 109.33) | 4.27 |  |

# Table S3. Experimentally investigated genes near QTLs for the number of panicles per plant identified with the main effect model.

| **Bin (chr, cM)***^a^* | **Gene/Location(chr, cM)** | **Distance (cM)***^b^* | **Reference** |
| --- | --- | --- | --- |
| 3(1, 0.72) | *OsJAG*/(1, 6.15) | 5.42 |  |
| 228(2, 8.57) | *OsTEF1*/(2, 13.87) | 5.30 |  |
|  | *qGY2-1*/(2, 17.51) | 8.94 |  |
| 353(2, 174.21) | *TH1*/(2, 174.45) | 0.24 |  |
| 461(3, 107.79) | *GS3*/(3, 94.72) | 13.08 |  |
|  | *OsPPKL1*/(3, 114.95) | 7.16 |  |
| 757(5, 61.38) | − | − | − |
| 818(5, 116.04) | *OsNADH*/(5, 106.49) | 9.55 |  |
| 861(6, 28.34) | *D3*/(6, 12.86) | 15.48 |  |
|  | *HGW*/(6, 15.77) | 12.57 |  |
| 908(6, 73.27) | *OsJMT1*/(6, 61.55) | 11.72 |  |
|  | *OsIAA23*/(6, 92.88) | 19.61 |  |
|  | *WAF1*/(7, 27.41) | 18.29 |  |
|  | *OsNRAMP5*/(7, 54.25) | 8.55 |  |
| 994(7, 45.70) | *OsTRXh1*/(7, 28.13) | 17.57 |  |
|  | *Ghd7*/(7, 54.73) | 9.03 |  |
|  | *RISBZ1*/(7, 28.13) | 17.57 |  |
| 1363(10, 53.62) | *Ehd1*/(10, 49.41) | 4.20 |  |
|  | *TAW1*/(10, 51.86) | 1.76 |  |

*^a^*Bin ID and the map information were obtained from dataset S1 in .

*^b^*Distance between the gene and the nearest QTL.

# Trait 2: the number of grains per panicle

# Table S4. Cross-validation for determining hyperparameters (*a*, *b*) used in QTL mapping for the number of grains per panicle.

| **Algorithm** | **Parameters (*a*, *b*)** | ***PE* ± *STE^a^*** |
| --- | --- | --- |
|
|  | (0.01, 0.01) | 261.48 ± 4.99 |
|  | (0.1, 0.1) | 239.04 ± 5.15 |
|  | (1, 1) | 271.73 ± 6.78 |
| EBlasso | (0.05, 0.1) | 238.84 ± 4.89*^b^* |
| Full model | (0.01, 0.1) | 239.05 ± 4.83 |
|  | (-0.01, 0.1) | 239.11 ± 4.82 |
|  | (-0.1, 0.1) | 240.32 ± 4.70 |
|  | (-0.3, 0.1) | 303.27 ± 10.05 |
|  | (0.05, 1) | 287.58 ± 5.67 |
|  | (0.05, 3) | 296.46 ± 5.58 |
|  | (0.01, 0.01) | 245.83 ± 10.58 |
|  | (0.1, 0.1) | 245.60 ± 10.63 |
|  | (1, 1) | 256.63 ± 14.89 |
| EBlasso | (0.1, 0.5) | 214.85 ± 13.61 |
| Main model | (-0.1, 0.5) | 211.22 ± 13.97 |
|  | (-0.3, 0.5) | 203.85 ± 14.01 |
|  | (-0.4, 0.5) | 197.25 ± 9.94*^b^* |
|  | (-0.5, 0.5) | 204.30 ± 9.50 |
|  | (-0.4, 1) | 200.30 ± 9.96 |
|  | (-0.4, 2) | 204.04 ± 9.69 |

*^a^*The average *PE* and standard error were obtained from five-fold cross validation.

*^b^*The optimal *PE* and its corresponding parameters used in QTL mapping.

# Table S5. Experimentally investigated genes near QTLs for the number of grains per panicle identified with the full model.

| **Bin (chr, cM)***^a^* | **Gene/Location(chr, cM)** | **Distance (cM)***^b^* | **Reference** |
| --- | --- | --- | --- |
| 10 (1, 6.15) | *OsJAG*/(1, 6.15) | 0.00 |  |
| 50 (1, 38.25) | *Gn1a*/(1, 30.72) | 7.53 |  |
|  | *OsAPC6*/(3, 22.66) | 17.98 |  |
| 381 (3, 40.64) | *D14*/(3, 37.67) | 2.97 |  |
|  | *PGL1*/(3, 35.14) | 5.50 |  |
| 436 (3, 93.03) | *GS3*/(3, 94.72) | 1.68 |  |
| 595 (4, 27.87) | − | − | − |
| 875 (6, 42.51) | *Hd1*/(6, 52.42) | 9.91 |  |
|  | *OsJMT1*/(6, 61.55) | 19.04 |  |
| 1004 (7, 54.01) | *Ghd7*/(7, 54.73) | 0.72 |  |
|  | *OsNRAMP5*/(7, 54.25) | 0.24 |  |
|  | *DEP2*/(7, 118.38) | 15.53 |  |
| 1057 (7, 133.92) | *OsSDR*/(7, 135.41) | 1.49 |  |
|  | *OsFAD8*/(7, 135.41) | 1.49 |  |
|  | *FZP*/(7, 135.41) | 1.49 |  |
|  | *SGL1*/(8, 88.50) | 10.58 |  |
| 1156 (8, 99.08) | *OsSPL14*/(8, 94.22) | 4.86 |  |
|  | *GW8*/(8, 106.89) | 7.81 |  |

*^a^*Bin ID and the map information were obtained from dataset S1 in .

*^b^*Distance between the gene and the nearest QTL.

# Table S6. Experimentally investigated genes near QTLs for the number of grains per panicle identified with the main effect model.

| **Bin (chr, cM)***^a^* | **Gene/Location(chr, cM)** | **Distance (cM)***^b^* | **Reference** |
| --- | --- | --- | --- |
| 43(1, 34.85) | *Gn1a*/(1, 30.72) | 4.13 |  |
| 436(3, 93.03) | *GS3*/(3, 94.72) | 1.69 |  |
| 877(6, 42.99) | *Hd1*/(6, 52.42) | 9.43 |  |
|  | *OsJMT1*/(6, 61.55) | 18.56 |  |
| 1006(7, 54.73) | *Ghd7*/(7, 54.73) | 0.00 |  |
|  | *OsNRAMP5*/(7, 54.25) | 0.48 |  |
|  | *DEP2*/(7, 118.38) | 15.54 |  |
| 1057(7, 133.92) | *OsSDR*/(7, 135.41) | 1.49 |  |
|  | *OsFAD8*/(7, 135.41) | 1.49 |  |
|  | *FZP*/(7, 135.41) | 1.49 |  |

*^a^*Bin ID and the map information were obtained from dataset S1 in .

*^b^*Distance between the gene and the nearest QTL.

# Trait 3: grain weight

# Table S7. Cross-validation for determining hyperparameters (*a*, *b*) used in QTL mapping for grain weight.

| **Algorithm** | **Parameters (*a*, *b*)** | ***PE* ± *STE^a^*** |
| --- | --- | --- |
|
|  | (0.01, 0.01) | 2.20 ± 0.01 |
|  | (0.1, 0.1) | 2.31 ± 0.02 |
|  | (1, 1) | 1.98 ± 0.02*^b^* |
| EBlasso | (0.1, 1) | 2.25 ± 0.03 |
| Full model | (0.01, 1) | 2.04 ± 0.02 |
|  | (-0.001, 1) | 2.14 ± 0.02 |
|  | (-0.01, 1) | 2.15 ± 0.01 |
|  | (-0.1, 1) | 2.17 ± 0.02 |
|  | (-0.2, 1) | 2.31 ± 0.03 |
|  | (1, 2) | 2.29 ± 0.04 |
|  | (0.01, 0.01) | 1.55 ± 0.03 |
|  | (0.05, 0.05) | 1.46 ± 0.05 |
|  | (0.1, 0.1) | 1.45 ± 0.06 |
| EBlasso | (0.5, 0.5) | 1.25 ± 0.02 |
| Main model | (1, 1) | 1.08 ± 0.03*^b^* |
|  | (0.5, 1) | 1.20 ± 0.07 |
|  | (0.1, 1) | 1.75 ± 0.10 |
|  | (0.01, 1) | 1.66 ± 0.03 |
|  | (-0.001, 1) | 1.75 ± 0.10 |
|  | (1, 2) | 1.60 ± 0.05 |

*^a^*The average *PE* and standard error were obtained from five-fold cross validation.

*^b^*The optimal *PE* and its corresponding parameters used in QTL mapping.

# Table S8. Experimentally investigated genes near QTLs of grain weight identified with the full model.

| **Bin group (chr, cM)***^a^* | **Gene/Location(chr, cM)** | **Distance (cM)***^b^* | **Reference** |
| --- | --- | --- | --- |
| 18,37(1, 12.06-31.68) | *OsJAG*/(1, 6.15) | 5.91 |  |
|  | *Gn1a*/(1, 30.72) | 0.97 |  |
| 67,71,96(1, 71.95-85.37) | − | − | − |
| 107,119(1, 99.35-105.68) | *OsEF3*/(1, 102.73) | 2.95 |  |
|  | *LOG*/(1, 113.27) | 7.59 |  |
|  | *DWARF10*/(1, 145.25) | 7.49 |  |
| 151,184(1, 137.77-154.78) | *LAX1*/(1, 136.09) | 1.68 |  |
|  | *D10*/(1, 145.25) | 7.49 |  |
| 210(1, 194.96) | − | − | − |
| 247(2, 33.83) | *qGY2-1*/(2, 17.51) | 16.32 |  |
|  | *OsTEF1*/(2, 13.87) | 19.96 |  |
| 310(2, 83.02) | *RCN2*/(2, 78.18) | 4.84 |  |
|  | *OsCIN1*/(2, 82.78) | 0.24 |  |
|  | *OsGS1*/(2, 146.32) | 1.21 |  |
| 329(2, 147.53) | *OsPIN1*/(2, 148) | 0.48 |  |
|  | *PGL2*/(2, 149.24) | 1.61 |  |
| 419,431(3, 82.45-89.56) | *GS3*/(3, 94.72) | 5.15 |  |
|  | *OsTB1*/(3, 134.05) | 0.73 |  |
| 472,500(3, 118.94-133.32) | *OsPPKL1*/(3, 114.95) | 3.99 |  |
|  | *OsTB1*/(3, 134.05) | 0.73 |  |
|  | *PAP2*/(3, 152.25) | 18.93 |  |
| 547(3, 187.25) | − | − | − |
| 583(4, 15.88) | − | − | − |
| 620(4, 53.86) | *GIF1*/(4, 53.86) | 0.00 |  |
|  | *LAX2*/(4, 49.32) | 4.54 |  |
|  | *D17*/(4, 84.20) | 0.00 |  |
| 647(4, 84.2) | *MIP1*/(4, 84.20) | 0.00 |  |
|  | *OsAP2-39*/(4,103.18) | 18.98 |  |
|  | *RFL*/(4, 96.82) | 12.63 |  |
|  | *SRS3*/(5, 20.04) | 4.98 |  |
|  | *OsPPKL2*/(5, 17.57) | 2.51 |  |
| 700,708,714(5, 8.70-15.06) | *OsSIZ1*/(5, 9.68) | 0.98 |  |
|  | *OsMT2b*/(5, 4.54) | 4.16 |  |
|  | *APG*/(5, 14.58) | 0.48 |  |

*^a^* Bin ID and the map information were obtained from dataset S1 in . QTLs with a distance ≤ 20cM are represented as one group.

*^b^*Distance between the gene and the nearest QTL.

**Table S8.** continued

| **Bin group (chr, cM)***^a^* | **Gene/Location(chr, cM)** | **Distance (cM)***^b^* | **Reference** |
| --- | --- | --- | --- |
| 727,729(5, 28.25-29.71) | *GS5*/(5, 20.52) | 7.73 |  |
|  | *qSW5*/(5, 33.28) | 3.57 |  |
| 772(5, 75.82) | − | − | − |
| 796,818(5, 104.57-116.04) | *OsNADH*/(5, 106.487) | 1.92 |  |
|  | *EUI1*/(5, 90.68) | 13.89 |  |
| 916,920(6, 78.19-81.17) | *OsJMT1*/(6, 61.55) | 19.62 |  |
|  | *OsIAA23*/(6, 92.88) | 11.71 |  |
| 955(6, 125.73) | *APO1*/(6, 122.81) | 2.92 |  |
|  | *OsPIN2*/(6, 122.81) | 2.92 |  |
|  | *LRK1*/(7, 8.62) | 2.18 |  |
|  | *PROG1*/(7, 25.67) | 14.87 |  |
| 971,987(7, 0.96-10.8) | *OsTRXh1*/(7, 28.13) | 17.33 |  |
|  | *WAF1*/(7, 27.41) | 16.61 |  |
|  | *RISBZ1*/(7, 28.13) | 17.33 |  |
| 1011(7, 60.93) | *Ghd7*/(7, 54.73) | 6.20 |  |
|  | *OsNRAMP5*/(7, 54.25) | 6.68 |  |
| 1026,1035(7, 82.71-101.53) | *DEP2*/(7, 118.38) | 16.86 |  |
|  | *DTH8*/(8, 36.74) | 1.68 |  |
| 1093,1100,1111,1117(8, 38.42-48.34) | *ASP1*/(8, 29.16) | 9.26 |  |
|  | *OsFIE2*/(8, 23.56) | 14.86 |  |
|  | *OsSPL14*/(8, 94.22) | 13.64 |  |
| 1167,1168(8, 107.86-110.43) | *GW8*/(8, 106.89) | 0.97 |  |
|  | *SGL1*/(8, 88.50) | 19.36 |  |
|  | *DEP1*/(9, 71.77) | 8.70 |  |
| 1224(9, 63.07) | *qPE9-1*/(9, 65.26) | 2.20 |  |
|  | *OsEATB*/(75.65) | 12.58 |  |
|  | *TAC1*/(9, 86.57) | 0.48 |  |
| 1262(9, 86.09) | *SG1*/(9, 80.28) | 5.82 |  |
|  | *LGD1*/(9, 86.57) | 0.48 |  |
| 1375(10, 74.22) | − | − | − |
|  | *RCN1*/(11, 17.19) | 2.73 |  |
| 1397,1400, 1407(11, 5.64-19.92) | *OsNAC5*/(11, 27.36) | 7.44 |  |
|  | *OsNAC10*/(11, 5.88) | 0.24 |  |
|  | *CycT1;3*/(11, 19.92) | 0.00 |  |
| 1461(11, 62.29) | *SRS5*/(11, 52.07) | 10.21 |  |
|  | *DWARF27*/(11, 100.57) | 4.79 |  |
| 1505(11, 95.79) | *TLD1*/(11, 83.27) | 12.52 |  |
|  | *D27*/(11, 101.79) | 6.01 |  |
| 1578(12, 55.85) | *OGR1*/(12, 42.95) | 12.90 |  |

# Table S9. Experimentally investigated genes near QTLs of grain weight identified with the main effect model.

| **Bin group (chr, cM)***^a^* | **Gene/Location(chr, cM)** | **Distance (cM)***^b^* | **Reference** |
| --- | --- | --- | --- |
| 37,38,50(1, 31.68 - 38.25) | *Gn1a*/(1, 30.72) | 0.96 |  |
|  | *LAX1*/(1, 136.09) | 1.68 |  |
| 151,173(1, 137.77 - 148.38) | *DWARF10*/(1, 145.25) | 3.13 |  |
|  | *D10*/(1, 145.26) | 3.12 |  |
| 199(1, 188.15) | − | *−* | *−* |
| 228(2, 8.57) | *OsTEF1*/(2, 13.87) | 5.30 |  |
|  | *qGY2-1*/(2, 17.51) | 8.94 |  |
|  | *OsPIP1;1*/(2, 136.02) | 0.73 |  |
| 312,332(2, 136.75 - 149.24) | *OsGS1*/(2, 146.32) | 2.92 |  |
|  | *OsPIN1*/(2, 148.00) | 1.24 |  |
|  | *PGL2*/(2, 149.24) | 0.00 |  |
| 440, 441(3, 93.99 - 94.23) | *GS3*/(3, 94.72) | 0.48 |  |
| 498(3, 132.60) | *OsTB1*/(3, 134.05) | 1.45 |  |
| 547(3, 187.25) | − | *−* | *−* |
|  | *OsPPKL2*/(5, 17.57) | 3.47 |  |
|  | *APG*/(5, 14.58) | 0.48 |  |
| 710,729(5, 14.10 - 29.71) | *OsSIZ1*/(5, 9.68) | 4.42 |  |
|  | *qSW5*/(5, 33.28) | 3.57 |  |
|  | *GS5*/(5, 20.52) | 6.42 |  |
|  | *SRS3*/(5, 20.04) | 5.94 |  |
|  | *D3*/(6, 12.86) | 0.96 |  |
| 843(6, 11.90) | *DLT*/(6, 5.82) | 6.08 |  |
|  | *HGW*/(6, 15.77) | 3.87 |  |
| 894(6, 58.14) | *Hd1*/(6, 52.42) | 5.72 |  |
|  | *OsJMT1*/(6, 61.55) | 3.40 |  |
|  | *MOC1*/(6, 98.75) | 5.87 |  |
| 936(6, 92.88) | *TGW6*/(6, 102.99) | 10.11 |  |
|  | *OsIAA23*/(6, 92.88) | 0.00 |  |
| 1008(7, 55.21) | *Ghd7*/(7, 54.73) | 0.48 |  |
| 1110(8, 44.70) | *DTH8*/(8, 36.74) | 7.96 |  |
|  | *ASP1*/(8, 29.16) | 15.54 |  |
| 1176(8, 120.35) | *GW8*/(8, 106.89) | 13.46 |  |

*^a^* Bin ID and the map information were obtained from dataset S1 in . QTLs with a distance ≤ 20cM are represented as one group.

*^b^*Distance between the gene and the nearest QTL.

**Table S9.** Continued

| **Bin group (chr, cM)***^a^* | **Gene/Location(chr, cM)** | **Distance (cM)***^b^* | **Reference** |
| --- | --- | --- | --- |
|  | *TAC1*/(9, 86.57) | 6.06 |  |
|  | *DEP1*/(9, 71.77) | 8.74 |  |
| 1251(9, 80.51) | *qPE9-1*/(9, 65.26) | 15.25 |  |
|  | *OsEATB*/(75.65) | 4.87 |  |
|  | *LGD1*/(9, 86.57) | 6.06 |  |
|  | *SG1*/(9, 80.28) | 0.23 |  |
| 1374(10, 73.98) | − | *−* | *−* |
|  | *SP1*/(11, 46.04) | 7.24 |  |
| 1442(11, 53.28) | *SRS5*/(11, 52.07) | 1.21 |  |
|  | *DWARF27*/(11, 100.57) | 4.29 |  |
| 1506(11, 96.27) | *TLD1*/(11, 83.27) | 13.00 |  |
|  | *D27*/(11, 101.79) | 5.52 |  |
| 1565(12, 45.60) | *OGR1*/(12, 42.95) | 2.65 |  |

# Trait 4: yield per plant

# Table S10. Cross-validation for determining hyperparameters (*a*, *b*) used in QTL mapping for yield per plant.

| **Algorithm** | **Parameters (*a*, *b*)** | ***PE* ± *STE^a^*** |
| --- | --- | --- |
|
|  | (0.01, 0.01) | 36.92 ± 0.83 |
|  | (0.1, 0.1) | 41.59 ± 1.20 |
|  | (1, 1) | 36.15 ± 0.81*^b^* |
| EBlasso | (0.5, 1) | 51.18 ± 0.94 |
| Full model | (0.1, 1) | 44.50 ± 0.74 |
|  | (0.05, 1) | 50.03 ± 0.86 |
|  | (0.01, 1) | 47.58 ± 0.79 |
|  | (-0.001, 1) | 47.42 ± 0.66 |
|  | (1, 2) | 42.91 ± 1.57 |
|  | (1, 3) | 48.14 ± 1.23 |
|  | (0.01, 0.01) | 31.75 ± 1.58 |
|  | (0.1, 0.1) | 31.73 ± 1.58 |
|  | (1, 1) | 32.82 ± 1.43 |
| EBlasso | (0.01, 0.1) | 31.74 ± 1.58 |
| Main model | (-0.01, 0.1 | 31.75 ± 1.58 |
|  | (-0.1, 0.1) | 31.66 ± 1.56 |
|  | (-0.3, 0.1) | 30.67 ± 1.46 |
|  | (-0.5, 0.1) | 29.33 ± 1.50*^b^* |
|  | (-0.5, 1) | 32.70 ± 2.00 |
|  | (-0.5, 2) | 31.15 ± 1.70 |

*^a^*The average *PE* and standard error were obtained from five-fold cross validation.

*^b^*The optimal *PE* and its corresponding parameters used in QTL mapping.

# Table S11. Experimentally investigated genes near QTLs of yield per plant identified with the full model.

| **Bin (chr, cM)***^a^* | **Gene/Location(chr, cM)** | **Distance (cM)***^b^* | **Reference** |
| --- | --- | --- | --- |
| 113 (1, 102.01) | *LOG*/(1, 113.27) | 11.26 |  |
|  | *OsEF3*/(1, 102.73) | 0.72 |  |
| 743 (5, 47.95) | *qSW5*/(5, 33.28) | 14.67 |  |
| 1014 (7, 63.67) | *OsNRAMP5*/(7, 54.25) | 9.42 |  |
|  | *Ghd7*/(7, 54.73) | 8.94 |  |
| 1043 (7, 116.17) | *DEP2*/(7, 118.38) | 2.21 |  |
|  | *FZP*/(7, 135.41) | 1.49 |  |
| 1057 (7, 133.92) | *OsSDR*/(7, 135.41) | 1.49 |  |
|  | *OsFAD8*/(7, 135.41) | 1.49 |  |
| 1144 (8, 64.09) | − | − | − |
| 1547 (12, 16.04) | − | − | − |

*^a^*Bin ID and the map information were obtained from dataset S1 in .

*^b^*Distance between the gene and the nearest QTL.

# Table S12. Experimentally investigated genes near QTLs of yield per plant identified with the main effect model.

| **Bin (chr, cM)***^a^* | **Gene/Location(chr, cM)** | **Distance (cM)***^b^* | **Reference** |
| --- | --- | --- | --- |
|  | *DWARF10*/(1, 145.25) | 7.83 |  |
| 181(1, 153.08) | *LAX1*/(1, 136.09) | 16.99 |  |
|  | *D10*/(1, 145.25) | 7.83 |  |
| 1014(7, 63.67) | *Ghd7*/(7, 54.73) | 8.94 |  |
|  | *OsNRAMP5*/(7, 54.25) | 9.42 |  |
|  | *DEP2*/(7, 118.38) | 15.54 |  |
| 1057(7, 133.92) | *FZP*/(7, 135.41) | 1.49 |  |
|  | *OsSDR*/(7, 135.41) | 1.49 |  |
|  | *OsFAD8*/(7, 135.41) | 1.49 |  |
| 1100(8, 42.06) | *DTH8*/(8, 36.74) | 5.32 |  |
|  | *ASP1*/(8, 29.16) | 12.90 |  |

*^a^*Bin ID and the map information were obtained from dataset S1 in .

*^b^*Distance between the gene and the nearest QTL.

# References

1. Duan Y, Diao Z, Liu H, Cai M, Wang F, et al. (2010) Molecular cloning and functional characterization of *OsJAG* gene based on a complete-deletion mutant in rice (*Oryza sativa L*.). Plant Mol Biol 74: 605-615.

2. Ashikari M, Sakakibara H, Lin S, Yamamoto T, Takashi T, et al. (2005) Plant science: Cytokinin oxidase regulates rice grain production. Science 309: 741-745.

3. Kurakawa T, Ueda N, Maekawa M, Kobayashi K, Kojima M, et al. (2007) Direct control of shoot meristem activity by a cytokinin-activating enzyme. Nature 445: 652-655.

4. Fu C, Yang X, Chen X, Chen W, Ma Y, et al. (2009) *OsEF3*, a homologous gene of *Arabidopsis* *ELF3*, has pleiotropic effects in rice. Plant Biol 11: 751-757.

5. Arite T, Iwata H, Ohshima K, Maekawa M, Nakajima M, et al. (2007) *DWARF10*, an *RMS1/MAX4/DAD1* ortholog, controls lateral bud outgrowth in rice. Plant J 51: 1019-1029.

6. Minakuchi K, Kameoka H, Yasuno N, Umehara M, Luo L, et al. (2010) *FINE CULM1* (*FC1*) works downstream of strigolactones to inhibit the outgrowth of axillary buds in rice. Plant Cell Physiol 51: 1127-1135.

7. Paul P, Awasthi A, Rai AK, Gupta SK, Prasad R, et al. (2012) Reduced tillering in Basmati rice T-DNA insertional mutant *OsTEF1* associates with differential expression of stress related genes and transcription factors. Funct Integer Genomic 12: 291-304.

8. He G, Luo X, Tian F, Li K, Zhu Z, et al. (2006) Haplotype variation in structure and expression of a gene cluster associated with a quantitative trait locus for improved yield in rice. Genome Res 16: 618-626.

9. Nakagawa M, Shimamoto K, Kyozuka J (2002) Overexpression of *RCN1* and *RCN2*, rice *TERMINAL FLOWER 1/CENTRORADIALIS* homologs, confers delay of phase transition and altered panicle morphology in rice. Plant J 29: 743-750.

10. Hirose T, Takano M, Terao T (2002) Cell wall invertase in developing rice caryopsis: molecular cloning of *OsCIN1* and analysis of its expression in relation to its role in grain filling. Plant Cell Physiol 43: 452-459.

11. Heang D, Sassa H (2012) An atypical bHLH protein encoded by *POSITIVE REGULATOR OF GRAIN LENGTH 2* is involved in controlling grain length and weight of rice through interaction with a typical bHLH protein APG. Breeding Sci 62: 133.

12. Xu M, Zhu L, Shou H, Wu P (2005) A *PIN1* family gene, *OsPIN1*, involved in auxin-dependent adventitious root emergence and tillering in rice. Plant Cell Physiol 46: 1674-1681.

13. Tabuchi M, Sugiyama K, Ishiyama K, Inoue E, Sato T, et al. (2005) Severe reduction in growth rate and grain filling of rice mutants lacking *OsGS1*;*1*, a cytosolic glutamine synthetase1;1. Plant J 42: 641-651.

14. Li X, Sun L, Tan L, Liu F, Zhu Z, et al. (2012) *TH1*, a *DUF640* domain-like gene controls lemma and palea development in rice. Plant Mol Biol 78: 351-359.

15. Moritoh S, Eun CH, Ono A, Asao H, Okano Y, et al. (2012) Targeted disruption of an orthologue of *DOMAINS REARRANGED METHYLASE 2*, *OsDRM2*, impairs the growth of rice plants by abnormal DNA methylation. Plant J 71: 85-98.

16. Kumar M, Basha PO, Puri A, Rajpurohit D, Randhawa GS, et al. (2010) A candidate gene *OsAPC6* of anaphase-promoting complex of rice identified through T-DNA insertion. Funct Integer Genomic 10: 349-358.

17. Lin Q, Wang D, Dong H, Gu S, Cheng Z, et al. (2012) Rice *APC/CTE* controls tillering by mediating the degradation of *MONOCULM 1*. Nat Commun 3: 752.

18. Xu C, Wang Y, Yu Y, Duan J, Liao Z, et al. (2012) Degradation of *MONOCULM 1* by *APC/CTAD1* regulates rice tillering. Nat Commun 3: 750.

19. Heang D, Sassa H (2012) Antagonistic actions of HLH/bHLH proteins are involved in grain length and weight in rice. PLoS ONE 7: e31325.

20. Mao H, Sun S, Yao J, Wang C, Yu S, et al. (2010) Linking differential domain functions of the *GS3* protein to natural variation of grain size in rice. Proc Natl Acad Sci USA 107: 19579-19584.

21. Zhang X, Wang J, Huang J, Lan H, Wang C, et al. (2012) Rare allele of *OsPPKL1* associated with grain length causes extra-large grain and a significant yield increase in rice. Proc Natl Acad Sci USA 109: 21534-21539.

22. Kobayashi K, Maekawa M, Miyao A, Hirochika H, Kyozuka J (2010) *PANICLE PHYTOMER2* (*PAP2*), encoding a SEPALLATA subfamily MADS-box protein, positively controls spikelet meristem identity in rice. Plant Cell Physiol 51: 47-57.

23. Li S, Zhao B, Yuan D, Duan M, Qian Q, et al. (2013) Rice zinc finger protein *DST* enhances grain production through controlling *Gn1a/OsCKX2* expression. Proc Natl Acad Sci USA 110: 3167-3172.

24. Ma X, Cheng Z, Qin R, Qiu Y, Heng Y, et al. (2013) *OsARG* encodes an arginase that plays critical roles in panicle development and grain production in rice. Plant J 73: 190-200.

25. Zhou G, Chen Y, Yao W, Zhang C, Xie W, et al. (2012) Genetic composition of yield heterosis in an elite rice hybrid. Proc Natl Acad Sci USA 109: 15847-15852.

26. Wang E, Xu X, Zhang L, Zhang H, Lin L, et al. (2010) Duplication and independent selection of cell-wall invertase genes *GIF1* and *OsCIN1* during rice evolution and domestication. BMC Evol Biol 10: 108.

27. Tabuchi H, Zhang Y, Hattori S, Omae M, Shimizu-Sato S, et al. (2011) *LAX PANICLE2* of rice encodes a novel nuclear protein and regulates the formation of axillary meristems. Plant Cell 23: 3276-3287.

28. Sun F, Zhang W, Xiong G, Yan M, Qian Q, et al. (2010) Identification and functional analysis of the *MOC1* interacting protein 1. J Genet Genomics 37: 69-77.

29. Rao NN, Prasad K, Kumar PR, Vijayraghavan U (2008) Distinct regulatory role for *RFL*, the rice *LFY* homolog, in determining flowering time and plant architecture. Proc Natl Acad Sci USA 105: 3646-3651.

30. She K-C, Kusano H, Koizumi K, Yamakawa H, Hakata M, et al. (2010) A novel factor *FLOURY ENDOSPERM2* is involved in regulation of rice grain size and starch quality. Plant Cell 22: 3280-3294.

31. Yaish MW, El-kereamy A, Zhu T, Beatty PH, Good AG, et al. (2010) The *APETALA-2*-like transcription factor *OsAP2-39* controls key interactions between abscisic acid and gibberellin in rice. PLoS Genet 6: e1001098.

32. Ishiyama K, Inoue E, Tabuchi M, Yamaya T, Takahashi H (2004) Biochemical background and compartmentalized functions of cytosolic glutamine synthetase for active ammonium assimilation in rice roots. Plant Cell Physiol 45: 1640-1647.

33. Shomura A, Izawa T, Ebana K, Ebitani T, Kanegae H, et al. (2008) Deletion in a gene associated with grain size increased yields during rice domestication. Nat Genet 40: 1023-1028.

34. Weng J, Gu S, Wan X, Gao H, Guo T, et al. (2008) Isolation and initial characterization of *GW5*, a major QTL associated with rice grain width and weight. Cell Res 18: 1199-1209.

35. Li Y, Fan C, Xing Y, Jiang Y, Luo L, et al. (2011) Natural variation in *GS5* plays an important role in regulating grain size and yield in rice. Nat Genet 43: 1266-1269.

36. Kitagawa K, Kurinami S, Oki K, Abe Y, Ando T, et al. (2010) A novel kinesin 13 protein regulating rice seed length. Plant Cell Physiol 51: 1315-1329.

37. Zhang Y, Zhu Y, Peng Y, Yan D, Li Q, et al. (2008) Gibberellin homeostasis and plant height control by *EUI* and a role for gibberellin in root gravity responses in rice. Cell Res 18: 412-421.

38. Tamura W, Kojima S, Toyokawa A, Watanabe H, Tabuchi-Kobayashi M, et al. (2011) Disruption of a novel *NADH-glutamate synthase2* gene caused marked reduction in spikelet number of rice. Front Plant Sci 2.

39. Tong H, Liu L, Jin Y, Du L, Yin Y, et al. (2012) *DWARF AND LOW-TILLERING* acts as a direct downstream target of a *GSK3/SHAGGY*-like kinase to mediate brassinosteroid responses in rice. Plant Cell 24: 2562-2577.

40. Ishikawa S, Maekawa M, Arite T, Onishi K, Takamure I, et al. (2005) Suppression of tiller bud activity in tillering dwarf mutants of rice. Plant Cell Physiol 46: 79-86.

41. Li J, Chu H, Zhang Y, Mou T, Wu C, et al. (2012) The rice *HGW* gene encodes a ubiquitin-associated (UBA) domain protein that regulates heading date and grain weight. PLoS ONE 7: e34231.

42. Endo-Higashi N, Izawa T (2011) Flowering time genes *Heading date 1* and *Early heading date 1* together control panicle development in rice. Plant Cell Physiol 52: 1083-1094.

43. Li X, Qian Q, Fu Z, Wang Y, Xiong G, et al. (2003) Control of tillering in rice. Nature 422: 618-621.

44. Ishimaru K, Hirotsu N, Madoka Y, Murakami N, Hara N, et al. (2013) Loss of function of the IAA-glucose hydrolase gene *TGW6* enhances rice grain weight and increases yield. Nat Genet 45: 707–711.

45. Jun N, Gaohang W, Zhenxing Z, Huanhuan Z, Yunrong W, et al. (2011) *OsIAA23*-mediated auxin signaling defines postembryonic maintenance of QC in rice. Plant J 68: 433-442.

46. Kim EH, Kim YS, Park S-H, Koo YJ, Do Choi Y, et al. (2009) Methyl jasmonate reduces grain yield by mediating stress signals to alter spikelet development in rice. Plant Physiol 149: 1751-1760.

47. Zha X, Luo X, Qian X, He G, Yang M, et al. (2009) Over-expression of the rice *LRK1* gene improves quantitative yield components. Plant Biotechnol J 7: 611-620.

48. Xue W, Xing Y, Weng X, Zhao Y, Tang W, et al. (2008) Natural variation in *Ghd7* is an important regulator of heading date and yield potential in rice. Nat Genet 40: 761-767.

49. Kawakatsu T, Yamamoto MP, Touno SM, Yasuda H, Takaiwa F (2009) Compensation and interaction between *RISBZ1* and *RPBF* during grain filling in rice. Plant J 59: 908-920.

50. Abe M, Yoshikawa T, Nosaka M, Sakakibara H, Sato Y, et al. (2010) *WAVY LEAF1*, an ortholog of Arabidopsis *HEN1*, regulates shoot development by maintaining MicroRNA and trans-acting small interfering RNA accumulation in rice. Plant Physiol 154: 1335-1346.

51. Wang Y, Li J (2008) Rice, rising. Nat Genet 40: 1273-1275.

52. Ishikawa S, Ishimaru Y, Igura M, Kuramata M, Abe T, et al. (2012) Ion-beam irradiation, gene identification, and marker-assisted breeding in the development of low-cadmium rice. Proc Natl Acad Sci USA 109: 19166-19171.

53. Zhang C-J, Zhao B-C, Ge W-N, Zhang Y-F, Song Y, et al. (2011) An apoplastic h-type thioredoxin is involved in the stress response through regulation of the apoplastic reactive oxygen species in rice. Plant Physiol 157: 1884-1899.

54. Li F, Liu W, Tang J, Chen J, Tong H, et al. (2010) Rice *DENSE AND ERECT PANICLE 2* is essential for determining panicle outgrowth and elongation. Cell Res 20: 838-849.

55. Nallamilli BRR, Zhang J, Mujahid H, Malone BM, Bridges SM, et al. (2013) Polycomb group gene *OsFIE2* regulates rice (*Oryza sativa*) seed development and grain filling via a mechanism distinct from *Arabidopsis*. PLoS Genet 9: e1003322.

56. Yoshida A, Ohmori Y, Kitano H, Taguchi-Shiobara F, Hirano H-Y (2012) *ABERRANT SPIKELET AND PANICLE1*, encoding a TOPLESS-related transcriptional co-repressor, is involved in the regulation of meristem fate in rice. Plant J 70: 327-339.

57. Wei X, Xu J, Guo H, Jiang L, Chen S, et al. (2010) *DTH8* suppresses flowering in rice, influencing plant height and yield potential simultaneously. Plant Physiol 153: 1747-1758.

58. Yan W-H, Wang P, Chen H-X, Zhou H-J, Li Q-P, et al. (2011) A major QTL, *Ghd8*, plays pleiotropic roles in regulating grain productivity, plant height, and heading date in rice. Mol Plant 4: 319-330.

59. Jiao Y, Wang Y, Xue D, Wang J, Yan M, et al. (2010) Regulation of *OsSPL14* by *OsmiR156* defines ideal plant architecture in rice. Nat Genet 42: 541-544.

60. Wang S, Wu K, Yuan Q, Liu X, Liu Z, et al. (2012) Control of grain size, shape and quality by *OsSPL16* in rice. Nat Genet 44: 950-954.

61. Nakagawa H, Tanaka A, Tanabata T, Ohtake M, Fujioka S, et al. (2012) *SHORT GRAIN1* decreases organ elongation and brassinosteroid response in rice. Plant Physiol 158: 1208-1219.

62. Huang X, Qian Q, Liu Z, Sun H, He S, et al. (2009) Natural variation at the *DEP1* locus enhances grain yield in rice. Nat Genet 41: 494-497.

63. Zhou Y, Zhu J, Li Z, Yi C, Liu J, et al. (2009) Deletion in a quantitative trait gene qPE9-1 associated with panicle erectness improves plant architecture during rice domestication. Genetics 183: 315-324.

64. Qi W, Sun F, Wang Q, Chen M, Huang Y, et al. (2011) Rice ethylene-response *AP2/ERF* factor *OsEATB* restricts internode elongation by down-regulating a gibberellin biosynthetic gene. Plant Physiol 157: 216-228.

65. Yu B, Lin Z, Li H, Li X, Li J, et al. (2007) *TAC1*, a major quantitative trait locus controlling tiller angle in rice. Plant J 52: 891-898.

66. Thangasamy S, Chen PW, Lai MH, Chen J, Jauh GY (2012) Rice *LGD1* containing RNA binding activity affects growth and development through alternative promoters. Plant J 71: 288-302.

67. Yoshida A, Sasao M, Yasuno N, Takagi K, Daimon Y, et al. (2013) *TAWAWA1*, a regulator of rice inflorescence architecture, functions through the suppression of meristem phase transition. Proc Natl Acad Sci USA 110: 767-772.

68. Song S-Y, Chen Y, Chen J, Dai X-Y, Zhang W-H (2011) Physiological mechanisms underlying *OsNAC5*-dependent tolerance of rice plants to abiotic stress. Planta 234: 331-345.

69. Jeong JS, Kim YS, Baek KH, Jung H, Ha S-H, et al. (2010) Root-specific expression of *OsNAC10* improves drought tolerance and grain yield in rice under field drought conditions. Plant Physiol 153: 185-197.

70. Qi P, Lin Y-S, Song X-J, Shen J-B, Huang W, et al. (2012) The novel quantitative trait locus GL3. 1 controls rice grain size and yield by regulating *Cyclin-T1;3*. Cell Res 22: 1666-1680.

71. Li S, Qian Q, Fu Z, Zeng D, Meng X, et al. (2009) *Short panicle1* encodes a putative *PTR* family transporter and determines rice panicle size. Plant J 58: 592-605.

72. Segami S, Kono I, Ando T, Yano M, Kitano H, et al. (2012) *Small and round seed 5* gene encodes alpha-tubulin regulating seed cell elongation in rice. Rice 5: 1-10.

73. Zhang S-W, Li C-H, Cao J, Zhang Y-C, Zhang S-Q, et al. (2009) Altered architecture and enhanced drought tolerance in rice via the down-regulation of indole-3-acetic acid by *TLD1/OsGH3*. 13 activation. Plant Physiol 151: 1889-1901.

74. Lin H, Wang R, Qian Q, Yan M, Meng X, et al. (2009) *DWARF27*, an iron-containing protein required for the biosynthesis of strigolactones, regulates rice tiller bud outgrowth. Plant Cell 21: 1512-1525.

75. Luan W, Liu Y, Zhang F, Song Y, Wang Z, et al. (2011) *OsCD1* encodes a putative member of the cellulose synthase-like D sub-family and is essential for rice plant architecture and growth. Plant Biotechnol J 9: 513-524.

76. Kim SR, Yang JI, Moon S, Ryu CH, An K, et al. (2009) Rice *OGR1* encodes a pentatricopeptide repeat–DYW protein and is essential for RNA editing in mitochondria. Plant J 59: 738-749.

77. Eom J-S, Cho J-I, Reinders A, Lee S-W, Yoo Y, et al. (2011) Impaired function of the tonoplast-localized sucrose transporter in rice, *OsSUT2*, limits the transport of vacuolar reserve sucrose and affects plant growth. Plant Physiol 157: 109-119.

78. Nair PMG, Kang I-S, Moon B-Y, Lee C-H (2009) Effects of low temperature stress on rice (*Oryza sativa* L.) plastid ω-3 desaturase gene, *OsFAD8* and its functional analysis using T-DNA mutants. Plant Cell 98: 87-96.

79. Kato T, Horibata A (2012) A novel frameshift mutant allele, *fzp-10*, affecting the panicle architecture of rice. Euphytica 184: 65-72.

80. Chuck G, Muszynski M, Kellogg E, Hake S, Schmidt RJ (2002) The control of spikelet meristem identity by the branched *silkless1* gene in maize. Science 298: 1238-1241.

81. Komatsu K, Maekawa M, Ujiie S, Satake Y, Furutani I, et al. (2003) *LAX* and *SPA*: major regulators of shoot branching in rice. Proc Natl Acad Sci USA 100: 11765-11770.

82. Zou J, Zhang S, Zhang W, Li G, Chen Z, et al. (2006) The rice *HIGH-TILLERING DWARF1* encoding an ortholog of Arabidopsis *MAX3* is required for negative regulation of the outgrowth of axillary buds. Plant J 48: 687-698.

83. Wang H, Makeen K, Yan Y, Cao Y, Sun S, et al. (2011) *OsSIZ1* regulates the vegetative growth and reproductive development in rice. Plant Mol Biol Rep 29: 411-417.

84. Yuan J, Chen D, Ren Y, Zhang X, Zhao J (2008) Characteristic and expression analysis of a metallothionein gene, *OsMT2b*, down-regulated by cytokinin suggests functions in root development and seed embryo germination of rice. Plant Physiol 146: 1637-1650.

85. Ikeda K, Ito M, Nagasawa N, Kyozuka J, Nagato Y (2007) Rice *ABERRANT PANICLE ORGANIZATION 1*, encoding an F-box protein, regulates meristem fate. Plant J 51: 1030-1040.

86. Chen Y, Fan X, Song W, Zhang Y, Xu G (2012) Over-expression of *OsPIN2* leads to increased tiller numbers, angle and shorter plant height through suppression of *OsLAZY1*. Plant Biotechnol J 10: 139-149.

87. Liu C, Fukumoto T, Matsumoto T, Gena P, Frascaria D, et al. (2013) Aquaporin *OsPIP1;1* promotes rice salt resistance and seed germination. Plant Physiol Bioch 63: 151-158.
